# Heteroligand Iron(V) Complexes Containing Porphyrazine, *trans*-Di[benzo]porphyrazine or Tetra[benzo]porphyrazine, Oxo and Fluoro Ligands: DFT Quantum-Chemical Study

**DOI:** 10.3390/ijms24076442

**Published:** 2023-03-29

**Authors:** Denis V. Chachkov, Oleg V. Mikhailov

**Affiliations:** 1 Kazan Department of Joint Supercomputer Center of Russian Academy of Sciences—Branch of Federal Scientific Center “Scientific Research Institute for System Analysis of the RAS”, Lobachevskii Street 2/31, 420111 Kazan, Russia; 2 Department of Analytical Chemistry, Certification and Quality Management, Kazan National Research Technological University, K. Marx Street 68, 420015 Kazan, Russia

**Keywords:** iron, heteroligand complex, oxo ligand, fluoro ligand, porphyrazine, *trans*-di[benzo]porphyrazine, tetra[benzo]-porphyrazine, phthalocyanine, DFT

## Abstract

By using quantum chemical calculation data obtained by the DFT method with the B3PW91/TZVP and OPBE/TZVP levels, the possibility of the existence of three Fe(V) complexes, each of which contains in the inner coordination sphere porphyrazine/*trans*-di[benzo]porphyrazine/tetra[benzo]porphyrazine (phthalocyanine), oxygen (O^2−^) and fluorine (F^−^) ions, was shown. Key geometric parameters of the molecular structure of these heteroligand complexes are given; it is noted that FeN4 chelate nodes, and all metal-chelate and non-chelate cycles in each of these compounds, are practically planar with the deviation from coplanarity, as a rule, by no more than 0.5°. Furthermore, the bond angles between two nitrogen atoms and an Fe atom are equal to 90°, or less than this by no more than 0.1°, while the bond angles between donor atoms N, Fe, and O or F, in most cases, albeit insignificantly, differ from this value. Nevertheless, the bond angles formed by Fe, O and F atoms are exactly 180°. It is shown that good agreement occurs between the structural data obtained using the above two versions of the DFT method. NBO analysis data for these complexes are presented; it is noted that, according to both DFT methods used, the ground state of the each of three complexes under consideration may be a spin quartet or spin doublet. Additionally, standard thermodynamic parameters of formation (standard enthalpy ∆*_f_H*^0^, entropy *S*^0^ and Gibbs’s energy ∆*_f_G*^0^) for the macrocyclic compounds under consideration are calculated.

## 1. Introduction

**Scheme 1:**
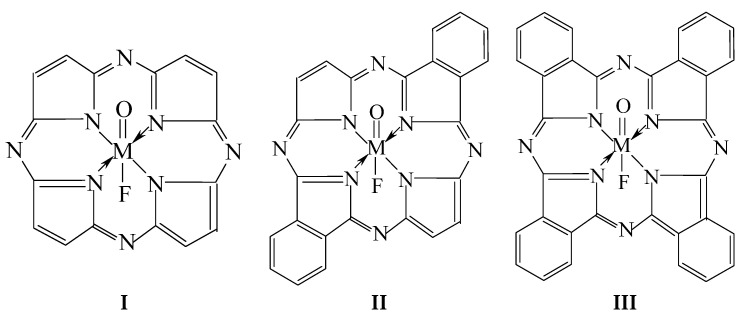
Structural formulas of complexes **I**, **II** and **III**.

In our previous articles [1,2], we carried out a quantum-chemical calculation of the molecular structures of coordination compounds having [M**L1**(O)F] (I), [M**L2**(O)F] (**II**) and [M**L3**(O)F] (**III**) formulas (M is Ni or Co, **L1**^2−^, **L2**^2−^ and **L3**^2−^ are double deprotonated forms of porphyrazine H_2_**L1**, *trans*-di[benzo]porphyrazine H_2_**L2** and tetra[benzo]porphyrazine (phthalocyanine) H_2_**L3**, respectively) (see Scheme 1).

According to the data presented in these works, obtained using two variants of the DFT method, namely B3PW91/TZVP and OPBE/TZVP, the possibility of the existence of all three types of complexes **I**–**III** in the case of M = Ni, but only one type, namely **III**, in the case of M = Co, was shown. Additionally, for both Ni and Co, the oxidation state in each of these compounds is equal to V (at least formally), which in both cases is the maximum among the reliably established values of this parameter for these chemical elements. The oxidation state of V is also very high for Fe, and to stabilize it with the participation of each of the above macrocyclic ligands, it is necessary that, in addition to these ligands, the inner coordination sphere of the complex contains ligands formed by the atoms with the highest electronegativity, specifically O^2−^ and F^−^ ligands. In this connection, it seems interesting to consider whether such complexes can be formed in the case of M = Fe, where the oxidation state V is intermediate between the minimum (–II) and maximum (VII) reliably established for this 3*d* element. It should be especially noticed that any information on iron compounds with **I**, **II** and **III** formulas, in the special literature devoted to macrocyclic ligands such as porphyrins, porphyrazines and their derivatives [3,4,5,6,7,8,9], or anywhere else, was not found by us. Nevertheless, complexes having these formulas are of rather considerable interest for the preparative chemistry of macrocyclic and coordination compounds. In addition, such substances may be helpful from a purely practical point of view, since, in principle, they can be used at least as catalysts for various organic synthesis processes and as components of specific redox systems. In this connection, the present paper was devoted to the consideration of the possibility of the existence of the complexes **I**, **II** and **III** using quantum-chemical calculation by density functional theory (DFT), which is now one of most popular methods of quantum chemistry and, in the case of a positive answer to this question, the calculation of the parameters of their molecular and electronic structures.

## 2. Results and Discussion

**Figure 1:**
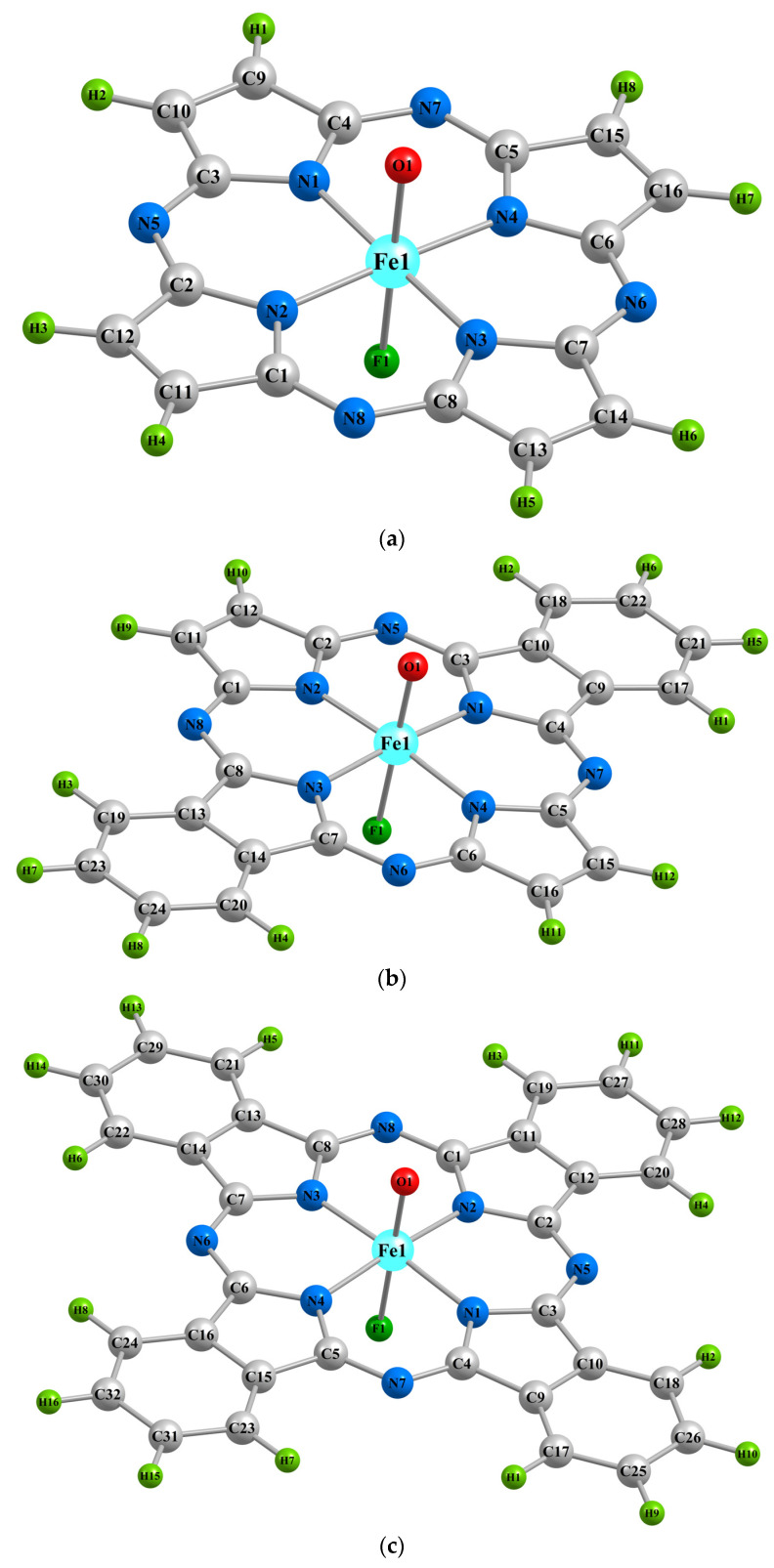
The images of molecular structures of the iron complexes **I**–**III** obtained as a result of quantum-chemical calculation by DFT B3PW91/TZVP: (**a**): [Fe**L1**(O)F], (**b**): [Fe**L2**(O)F], (**c**): [Fe**L3**(O)F].

It should be immediately noticed that, according to the data obtained with each of the DFT quantum chemical methods used here, for the 3d element under consideration, the formation of all three types of complexes **I**–**III** indicated above takes place. The lengths of chemical bonds between various atoms and bond angles for [Fe**L1**(O)F], [Fe**L2**(O)F] and [Fe**L3**(O)F] coordination compounds under study calculated using each of the DFT versions indicated above are given in Table 1. The images of molecular structures of these compounds obtained by the DFT B3PW91/TZVP method are shown in Figure 1, obtained by the DFT OPBE/TZVP method, in Figure S1 (see Supplementary Materials). As can be seen from Table 1, both variants of the DFT method, B3PW91/TZVP and DFT OPBE/TZVP, not only predict a stable molecular structure for all three compounds **I**, **II** and **III** in the case M = Fe, but also give quantitative characteristics of their molecular structures that differ only slightly from each other. Regarding the lengths of bonds between iron and nitrogen atoms, oxygen and fluorine, to which a given metal atom is bonded in these macrocyclic complexes, it may be concluded that the iron–oxygen bonds are the shortest, the iron–nitrogen bonds are the longest, and the iron–fluorine bonds occupy an intermediate position. By taking into account the radii of Fe, O, N and F atoms, it can be argued that the values of the above-mentioned bond lengths given in Table 1 correspond to the lengths of the double bond Fe=O and the single bonds Fe–F and Fe–N. It is characteristic that the Fe–N bonds in each of compounds **I**–**III** are equal only in pairs (Table 1). This difference is quite understandable, because in their molecular structures, the N1 and N3 atoms, strictly speaking, are not equivalent to the N2 and N4 atoms. The lengths of Fe–N bonds in the [Fe**L1**(O)F]-[Fe**L2**(O)F]-[Fe**L3**(O)F] series generally increase, while the lengths of the Fe=O and Fe–F bonds decrease. Such a circumstance may be associated, on the one hand, with an increase in the size of the “chelate cell” in the macrocyclic compounds under study, and, on the other hand, with the magnification of the positive charge on the iron atom upon passing from the **L1**^2−^ ligand to the **L3**^2−^ ligand. In this regard, it should be noted that exactly the same situation with respect to the lengths of the above bonds also takes place in nickel complexes similar in composition, as described in [1]. FeN_4_ chelate nodes and all four six-membered metal chelate rings as well as all four five-membered non-chelate ones that contain one nitrogen and four carbon atoms and adjacent to six-membered metal chelate rings in the metal macrocyclic compounds **I**, **II** and **III** are strong or practically strong planar, since the sums of the bond angles in each of these structural fragments (***BAS***, NBAS, BAS^6^ and BAS^5^) are 360.0°, 360°, 720.0° and 540.0°, respectively, or values close to these, differing from the indicated values, as a rule, by no more than 0.5°. The ***BAS*** values in the MN_4_ chelate nodes of all these coordination compounds are practically the same, and all bond angles (NFeN) are the same in them, which, although quite a bit, still differ from 90° (Table 1). As for the non-bond angles (NNN) in the N_4_ group, these angles according to the DFT B3PW91/TZVP method data, are equal only in pairs in each of the compounds **I**–**III**; according the DFT OPBE/TZVP method data, such pairwise equality takes place only in the case of complex **II**, in complexes **I** and **III**, all angles (NNN) are equal to 90° (Table 1). The six-membered chelate rings in each of these complexes are the same, both in terms of the sum of bond angles and their sets. For five-membered rings, this similarity takes place only in complexes **I** and **III**, and only within the framework of the DFT OPBE/TZVP method; according to the DFT B3PW91/TZVP method, they differ from each other in all three of these complexes. For the [Fe**L2**(O)F] complex, this difference is quite understandable, given that two of these four rings are linked to six-membered “phenylene” groups, while the other two are not linked. However, why all five-membered rings within the DFT B3PW91/TZVP method are different not only in the [Fe**L2**(O)F] complex, but also in the [Fe**L1**(O)F] and [Fe**L3**(O)F] complexes, remains unclear. The atoms of oxygen, iron, and fluorine in all the complexes under discussion form bond angles equal to or almost equal to 180°. It is noteworthy that a similar situation is also observed in the Co(V) and Ni(V) complexes of the same type considered in [1,2]. What is also noteworthy is that none of the bond angles (OFeN) and (FFeN) formed by Fe atom, donor N, O and F atoms is equal to 90°, despite the fact that the FeN_4_ chelate node in each of these metal macrocyclic complexes is almost coplanar. In addition, these bond angles are equal to each other only in pairs (Table 1); this also takes place in all analogous nickel complexes [1]. In cobalt complex of the type **III**, these angles are the same, although none of them is equal to 90° [2]. These data, as well as the fact that the above bond lengths are different from each other, make it possible to attribute the iron complexes under examination to the number of pseudo-octahedral complexes having tetragonal distortion, whereby the carbon–nitrogen and carbon–carbon bond lengths in both chelate and non-chelate cycles in each of complexes **I**–**III**, calculated by the above two DFT methods, are very close to each other. In this way, it can be argued that the molecular structures of each of the complexes [Fe**L1**(O)F], [Fe**L2**(O)F] and [Fe**L3**(O)F], found by both DFT methods used, show a very significant similarity to each other, not only qualitatively, but also quantitatively.

The values of the dipole electric moments (μ) for the given complexes obtained using DFT B3PW91/TZVP are 0.85, 0.42 and 0.42 Debye units; using DFT OPBE/TZVP, they are 0.78 ([Fe**L1**(O)F]), 0.35 ([Fe**L2**(O)F]) and 0.67 ([Fe**L3**(O)F]) Debye units, respectively. As can be seen, they differ noticeably from zero, which is quite understandable, since there is no center of symmetry in these complexes. However, they are not too significant and, on the whole, they are quite consistent with the concept of an almost planar structure of macrocyclic fragments **L1**^2−^, **L2**^2−^ and **L3**^2−^ which are part of the complexes under study. The numerical values of this parameter calculated by these two DFT methods, as well as the parameters of molecular structures, do not generally differ too much from each other. The exceptions, however, are the values (μ) for the [Fe**L3**(O)F] complex, which differ from each other by more than one and a half times. We believe that the results found by the DFT B3PW91/TZVP are more reliable, since the DFT method variants that use the B3PW91 functional describe the parameters of molecular structures better than the DFT versions that use the OPBE functional.

Key NBO analysis data, and, namely, the values of effective charges on the central iron atom and the nitrogen, oxygen and fluorine ones bonded with the central atom for the macrocyclic iron compounds **I**–**III** obtained by both DFT versions indicated above are presented in Table 2. Complete NBO data for all these complexes are given in the Supplementary Materials. As can be seen from these data, the values of the charges on individual atoms are very different from those that they would have if all the chemical bonds in these compounds were of a purely ionic nature; this fact, in our opinion, directly indicates a very high degree of electron density delocalization in the metal complexes considered here.

According to DFT B3PW91/TZVP calculation data, the ground state of the complexes **II** and **III** is spin quartet (*M_S_* = 4); additionally, the nearest excited state having *M_S_* value different from spin multiplicity of the ground state, namely spin doublet, is higher than the ground state on 49.9 kJ/mol for [Fe**L2**(O)F] and on and 59.0 kJ/mol for [Fe**L3**(O)F]. The calculation by the DFT OPBE/TZVP method indicates that the ground state of complexes **I** and **II** is a spin quartet, too; the nearest excited triplet is higher on 5.5 and 2.7 kJ/mol, respectively. This conclusion is also supported by the <S**2> (operator of the square of the intrinsic angular momentum of the total spin of the system) values, which are in the range of (3.75–3.85), and corresponds to the presence of three unpaired electrons in each of these complexes and configurations 3*d*^3^. However, in the case of complex **I**, according to DFT B3PW91/TZVP, and of complex **III**, according to the DFT OPBE/TZVP method, another situation takes place—the ground state is a spin doublet, and the nearest excited quartet state is higher, on 9.4 and 1.1 kJ/mol, respectively. In both cases, the <S**2> values (1.6043 in the case of complex I and 1.1656 in the case of complex **III**) are intermediate between the values of this parameter, which correspond to the presence of one and three unpaired electrons in the system, namely 0.7500 and 3.7500, where testing the wave functions of the ground and excited states for stability within each of these methods by using the standard STABLE = OPT procedure showed that the wave function of each states indicated above was stable under the considered perturbations for the each of complexes **I**, **II** and **III** under examination. In this connection, an unambiguous conclusion about the spin multiplicity of the ground state can be drawn from the data of these calculations only for complex **II**; in the case of complexes **I** and **III**, additional studies on this problem are needed. However, for complex **II**, the NBO analysis results calculated by the DFT B3PW91/TZVP and DFT OPBE/TZVP methods differ quite noticeably from each other (Table 2; see also the full NBO analysis data presented in Supplementary Materials). On the other hand, noteworthy is the very small difference between the energies of the ground and nearest excited states (<10 kJ/mol), which is observed in four out of six cases (in complex **I**, in the framework of both methods; in complex **II**, in the framework of the DFT OPBE/TZVP method; in complex **III**, within the framework of the DFT B3PW91/TZVP method). In this regard, there are certain grounds for believing that, at least in the case of the [Fe**L2**(O)F] complex, such a specific phenomenon as spin crossover is possible. 

**Figure 2:**
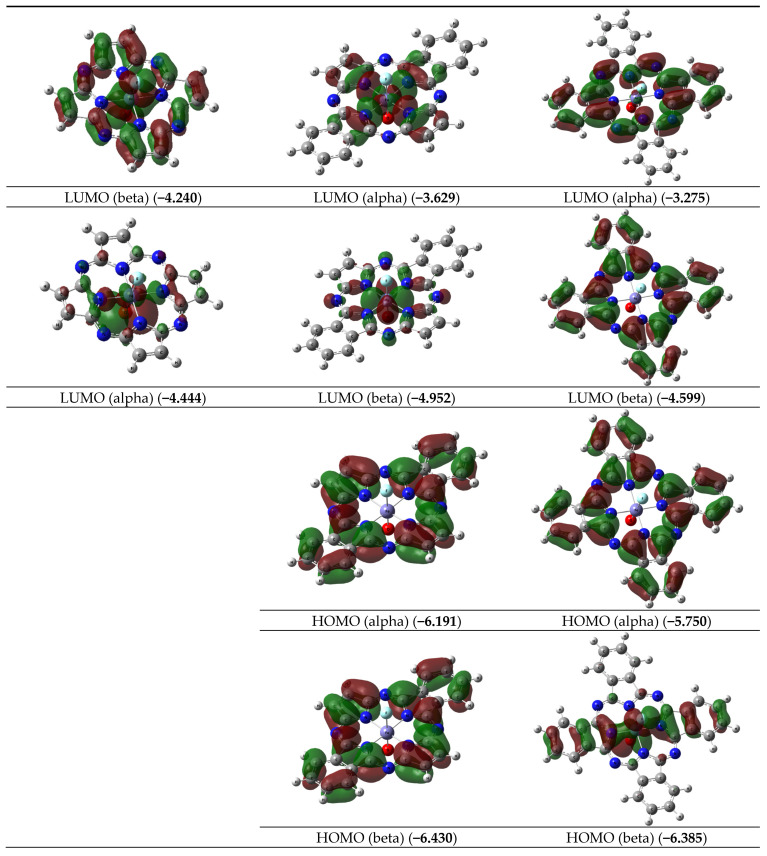

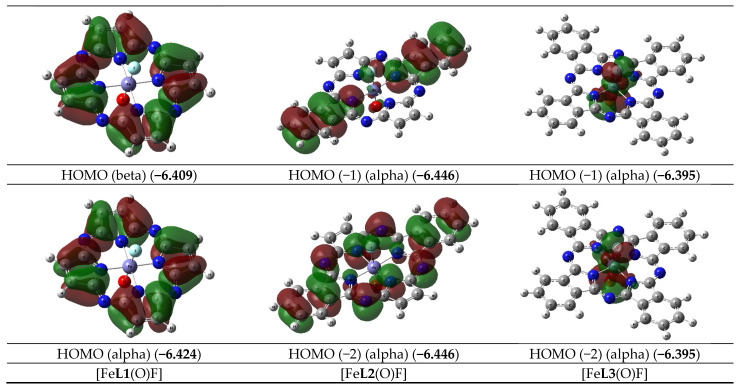
The images of HOMO and LUMO in the Fe**L1**(O)F] (ground state–spin doublet, *M_S_* = 2), Fe**L2**(O)F] and [Fe**L3**(O)F] (ground state–spin quartet, *M_S_* = 4) according to the DFT B3PW91/TZVP method. The energies values of the indicated MOs (in brackets) are given in eV. The symbol “alpha” belongs to electron with spin (+1/2), symbol “beta”, to electron with spin (−l/2). The abbreviations “HOMO (−1) (alpha)” and “HOMO (−2) (alpha)” are used to designate MOs located slightly below HOMO (alpha) and closest to it in order of decreasing energy values.

Images of the highest occupied (HOMO) and lowest vacant (LUMO) molecular orbitals of the considered complexes Fe**L1**(O)F], [Fe**L2**(O)F] and [Fe**L3**(O)F], obtained using DFT B3PW91/TZVP and DFT OPBE/TZVP methods are shown in Figure 2 and Figure S2 (see Supplementary Materials), respectively. As can be seen from these, both the shapes of HOMO and LUMO and the values of their energies calculated by these versions of the DFT method differ quite significantly from each other. In this case, which is characteristic, in the series [Fe**L1**(O)F]-[Fe**L2**(O)F]-[Fe**L3**(O)F] the energies of both HOMO and LUMO increase; as a rule, the lowest among them is HOMO (beta), the highest—LUMO (alpha).

The standard thermodynamic parameters of formation (∆*_f_H*^0^, *S*^0^ and ∆*_f_G*^0^) for the iron compounds **I**, **II** and **III** are presented in Table 3. As can be easily noted, the values of both ∆*_f_H*^0^ and ∆*_f_G*^0^ are positive for the each of [Fe**L1**(O)F], [Fe**L2**(O)F] and [Fe**L3**(O)F] complexes. Hence, none of these complexes can be obtained from simple substances formed by chemical elements in their compositions (and namely carbon, nitrogen, oxygen, fluorine and iron). Nonetheless, according to the data obtained as a result of the quantum-chemical calculations carried out by two independent methods of density functional theory, OPBE/TZVP and B3PW91/TZVP, all three iron complexes—types **I–III**—considered in this given article would be able to act as individual chemical compounds, at least in the gas phase, whereby it is typical, in the series [Fe**L1**(O)F]-[Fe**L2**(O)F]-[Fe**L3**(O)F], that the values of ∆*_f_H*^0^ and ∆*_f_G*^0^ decrease; this fact at least indirectly indicates an increase in their resistance to break down into simpler components.

## 3. Methods and Materials

As in our articles [1,2] cited above, as well as in previous ones, e.g., [10,11,12], the version of the DFT method with B3PW91/TZVP level combining the TZVP extended triple zeta split-valence basis set and B3PW91 functional [13,14] was used. According to data [15], this functional had a minimal value of so-called “normal error” in comparison with other DFT versions. Such an output is confirmed by comparing the data of calculating the structural parameters of 3*d*-element macrocyclic coordination compounds with phthalocyanine, obtained using different versions of the DFT method, with the experimental values of these parameters. Additionally, for comparison as in our articles [16,17,18,19,20,21,22], quantum-chemical calculations were carried out using the DFT method with OPBE/TZVP level combining TZVP basis set [23,24] and the OPBE functional [25,26]. As shown in [26,27,28,29,30], for the 3*d* elements, this variant of the DFT method more adequately predicts the relative energy stabilities of high-spin and low-spin states, and also reliably describes the most important geometric parameters of corresponding molecular structures. Calculations are performed with the Gaussian09 program package [31]. The correspondence of the discovered stationary points to energy minima was proved in all cases by the calculation of energy second derivatives with respect to atom coordinates; all equilibrium structures corresponding to minima of the potential energy surfaces had only real positive frequency values. Theoretically, Fe(V), which is in the complexes **I**–**III**, must have 3*d*^3^ electronic configuration, and that is why spin multiplicities 2 and 4 were considered for the given central ion in the course of calculation. Among the structures optimized at these multiplicities, the lowest-lying structure was selected. Parameters of molecular structures with the given multiplicities were calculated using the unrestricted (*UB3PW91*, *UOPBE*) method. The energetically most favorable structure has always been checked using the STABLE = OPT procedure, whereby the wave function corresponding to this structure was stable in all cases. Natural Bond Orbital (NBO) analysis was carried out using NBO version 3.1, integrated into the Gaussian09 program [31] according to the methodology described in [32]. NBO methods are well known for their excellent numerical stability and convergence with respect to basis set expansion, and for being sensibly proportionate to convergence of energy and other calculated wavefunction properties (unlike Mulliken analysis and related overlap-dependent methods in this case). The standard thermodynamic parameters of a formation (∆*_f_H*^0^, *S*^0^ and ∆*_f_G*^0^) for the metal macrocyclic compounds under consideration were calculated using the method described in [33].

## 4. Conclusions

Therefore, the data obtained using the DFT methods with OPBE/TZVP and B3PW91/TZVP levels and presented in this article are sufficiently reliable to confirm the principal possibility of the existence, as a minimum, of three novel Fe(V) macrocyclic compounds having [Fe**L1**(O)F], [Fe**L2**(O)F] and [Fe**L3**(O)F] compositions, where **L1**^2−^, **L2**^2−^ and **L3**^2−^ are, respectively, a double deprotonated form of porphyrazine H_2_**L1**, *trans*-di[benzo]porphyrazine H_2_**L2** and tetra[benzo]porphyrazine H_2_**L3**. In the each of these complexes, there are five chemical bonds formed by Fe atoms with atoms having greater electronegativity than Fe, according to the exchange mechanism—two bonds with N atoms, two bonds with O atom and one with an F atom. According to the definition of the term “oxidation degree” presented in [20], we may assume (of course, with a certain degree of caution) that the oxidation degree of iron atoms in the [Fe**L1**(O)F], [Fe**L2**(O)F] and [Fe**L3**(O)F] macrocyclic compounds under study might be equal to +5, and hence, the oxidation state of iron in them is V. It should be noted in this connection that such an oxidation state is uncharacteristic for iron and was noted only in a small number of works (see, in particular, [34,35,36,37]). However, there is still no information on iron complexes containing any porphyrin and porphyrazine derivatives in the inner coordination sphere [8,9]. Of course, the real charge on the iron atoms in any of these compounds differs significantly from the value of +5.00 ē; however, the given parameter has not been connected directly with the definition of oxidation degree and, if so, then it, in principle, cannot be used for the definition of oxidation state [38], whereby the results of our quantum-chemical calculations using both of the above variants of the DFT method, namely B3PW91/TZVP and OPBE/TZVP, are quite consistent with the concept of a rather high stability of those complexes with a high degree of oxidation of the central atom of the 3d element in the inner coordination sphere of which are acido ligands such as F^−^ and O_2_^−^ containing atoms of chemical elements with the highest electronegativity [38,39,40].

Nevertheless, at the present, it is expedient to confirm the possibility of the existence of compounds **I**, **II** and **III** experimentally, because their synthesis might be important for further chemistry development of both the highest oxidation states of d-elements, and, of course, of the chemistry of iron. Judging by the voluminous data presented in the review article [36], as well as in original articles [35,37], iron complexes containing close to **L1**, **L2**, and **L3** (NNNN) donor-atomic polydentate ligands with high oxidation states for this element (namely, Fe(IV), Fe(V) and Fe(VI)) can be used as potential catalysts; taking these data into account, it seems very likely that the macrocyclic metal chelates [Fe**L1**(O)F], [Fe**L2**(O)F] and [Fe**L3**(O)F] in this regard will also be quite promising. Perhaps (and even very likely) these compounds can be catalysts in other practically important reactions of organic synthesis (carbonylation, dehydrogenation, oxidation, etc.). It can be assumed that these compounds, in addition to their use in catalysis, are capable of finding their application as components of sensory and redox systems, specific agents in biochemical processes, and drugs in the treatment of various diseases. The latter is all the more likely if we take into account the important circumstance that the each of complexes **I**–**III** must be very strong oxidizing agents and, therefore, must also be potent antibacterial and antiviral agents. In addition, predicting the existence possibility of the exotic coordination compounds and modeling their molecular structures using modern quantum chemical calculations (and, in particular, the DFT methods of various levels) is a very useful tool in solving problems associated with such a synthesis. Now it is up to us to confirm the possibility of the existence of these exotic macrocyclic metal complexes in a real chemical experiment.

## Figures and Tables

**Table 1:** Bond lengths and bond angles in the iron macrocyclic compounds [Fe**L1**(O)F], [Fe**L2**(O)F] and [Fe**L3**(O)F] calculated by DFT B3PW91/TZVP and DFT OPBE/TZVP methods.

Macrocyclic Compound	[FeL1(O)F]		[FeL2(O)F]		[FeL3(O)F]	
Parameter of molecular structure	Calculated by DFT		Calculated by DFT		Calculated by DFT	
	B3PW91/TZVP	OPBE/TZVP	B3PW91/TZVP	OPBE/TZVP	B3PW91/TZVP	OPBE/TZVP
The lengths of Fe–N bonds in MN_4_ chelate node, *pm*						
Fe1N1	192.7	193.0	194.6	194.5	195.3	195.3
Fe1N2	193.7	193.0	193.8	193.6	195.3	195.3
Fe1N3	192.7	193.0	194.6	194.5	195.3	195.3
Fe1N4	193.7	193.0	193.8	193.6	195.3	195.3
The lengths of Fe–O and Fe–F bonds, *pm*						
Fe1O1	172.2	167.0	165.4	166.9	165.4	163.7
Fe1F1	178.8	187.4	188.4	187.8	188.1	186.2
The lengths of C–N bonds in 6-numbered chelate rings, *pm*						
N1C3	136.5	136.6	135.7	136.7	135.6	136.4
N1C4	136.5	136.6	135.7	136.7	135.6	136.4
N2C1	136.0	136.5	135.1	136.2	135.6	136.5
N2C2	136.0	136.5	135.1	136.2	135.6	136.5
N3C7	136.5	136.6	135.7	136.7	135.6	136.4
N3C8	136.5	136.6	135.7	136.7	135.6	136.3
N4C5	136.0	136.5	135.1	136.2	135.6	136.3
N4C6	136.0	136.5	135.1	136.2	135.6	136.5
N5C2	131.9	132.1	132.0	132.2	131.8	132.2
N5C3	131.9	132.1	132.1	131.9	131.8	132.1
N6C6	131.9	132.1	132.0	132.2	131.8	132.1
N6C7	131.9	132.1	132.1	131.9	131.8	132.2
N7C4	131.9	132.1	132.1	131.9	131.8	132.1
N7C5	131.9	132.1	132.0	132.2	131.8	132.2
N8C1	131.9	132.1	132.0	132.2	131.8	132.1
N8C8	131.9	132.1	132.1	131.9	131.8	132.2
The lengths of C–C bonds in 5-numbered chelate ring (N1C4C9C10C3), *pm*						
C4C9	144.6	145.4	146.4	146.0	146.4	146.1
C9C10	135.3	135.5	139.6	140.2	139.6	140.2
C10C3	144.6	145.4	146.4	146.0	146.4	146.1
Bond angles in chelate node FeN_4_, *deg*						
(N1Fe1N2)	90.0	89.9	89.9	89.9	89.9	89.9
(N2Fe1N3)	90.0	89.9	89.9	89.9	89.9	89.9
(N3Fe1N4)	90.0	89.9	89.9	89.9	89.9	89.9
(N4Fe1N1)	90.0	89.9	89.9	89.9	89.9	89.9
Bond angles sum (*BAS*), *deg*	**360.0**	**359.6**	**359.6**	**359.6**	**359.6**	**359.6**
Non-bond angles between N atoms in N_4_ grouping, *deg*						
(N1N2N3)	89.7	90.0	90.2	90.3	90.0	90.0
(N2N3N4)	90.3	90.0	89.8	89.7	90.0	90.0
(N3N4N1)	89.7	90.0	90.2	90.3	90.0	90.0
(N4N1N2)	90.3	90.0	89.8	89.7	90.0	90.0
Non-bond angles sum (NBAS), *deg*	360.0	360.0	360.0	360.0	360.0	360.0
Bond angles in 6-numbered chelate ring (Fe1N1C4N7C5N4), *deg*						
(Fe1N1C4)	126.0	126.0	125.5	125.3	125.5	125.6
(N1C4N7)	128.0	127.3	128.2	127.7	128.2	128.3
(C4N7C5)	122.1	122.3	122.2	122.2	122.6	122.2
(N7C5N4)	127.8	127.4	127.9	127.3	128.2	128.3
(C5N4Fe1)	126.1	126.2	126.2	125.8	125.5	125.6
(N4Fe1N1)	90.0	89.9	89.9	89.9	89.9	89.9
Bond angles sum (BAS^61^), *deg*	720.0	719.1	719.9	718.2	719.9	719.9
Bond angles in 6-numbered chelate ring (Fe1N4C6N6C7N3), *deg*						
(Fe1N4C6)	126.1	126.2	126.2	125.8	125.5	125.6
(N4C6N6)	127.8	127.4	127.9	127.3	128.2	128.3
(C6N6C7)	122.1	122.3	122.2	122.2	122.6	122.2
(N6C7N3)	128.0	127.3	128.2	127.7	128.2	128.3
(C7N3Fe1)	126.0	126.0	125.5	125.3	125.5	125.6
(N3Fe1N4)	90.0	89.9	89.9	89.9	89.9	89.9
Bond angles sum (BAS^62^), *deg*	720.0	719.1	719.9	718.2	719.9	719.9
Bond angles in 5-numbered ring (C3N1C4C9C10), *deg*						
(C3N1C4)	107.9	107.2	109.2	108.8	109.0	108.8
(N1C4C9)	108.9	109.5	109.3	109.3	109.4	109.4
(C4C9C10)	107.2	106.9	106.1	106.3	106.1	106.2
(C9C10C3)	107.1	106.9	106.1	106.3	106.1	106.2
(C10C3N1)	108.9	109.5	109.3	109.3	109.4	109.4
Bond angles sum (BAS^51^), *deg*	540.0	540.0	540.0	540.0	540.0	540.0
Bond angles in 5-numbered ring (C1N2C2C12C11), *deg*						
(C1N2C2)	107.8	107.2	107.6	107.2	109.0	108.8
(N2C2C12)	109.1	109.5	109.6	109.7	109.4	109.4
(C2C12C11)	107.0	106.9	106.6	106.7	106.1	106.2
(C12C11C1)	107.0	106.9	106.6	106.7	106.1	106.2
(C11C1N2)	109.1	109.5	109.6	109.7	109.4	109.4
Bond angles sum (BAS^51^), *deg*	540.0	540.0	540.0	540.0	540.0	540.0
Bond angles between O, Fe and N atoms, *deg*						
O1Fe1N1	89.4	92.7	92.6	91.8	92.6	92.3
O1Fe1N2	91.2	91.8	92.7	92.6	92.6	92.6
O1Fe1N3	89.4	92.7	92.6	91.8	92.6	92.2
O1Fe1N4	91.2	91.8	92.7	92.6	92.6	92.6
Bond angles between F, Fe and N atoms, *deg*						
F1Fe1N1	90.6	87.3	87.4	88.2	87.4	87.8
F1Fe1N2	88.8	88.2	87.3	87.4	87.4	87.4
F1Fe1N3	90.6	87.3	87.4	88.2	87.4	87.7
F1Fe1N4	88.8	88.2	87.3	87.4	87.4	87.4
Bond angles between O, Fe and F atoms, *deg*						
O1Fe1F1	180.0	180.0	180.0	180.0	180.0	179.9

**Table 2:** NBO analysis data for the iron compounds [Fe**L1**(O)F], [Fe**L2**(O)F] and [Fe**L3**(O)F] calculated by the DFT B3PW91/TZVP and DFT OPBE/TZVP methods.

Macrocyclic Compound	DFT Level	Effective Charge on Atom, in Electron Charge Units (ē)					<S**2>
		Fe1	N1 (N3)	N2 (N4)	O1	F1	
[Fe**L1**(O)F]	OPBE/TZVP	+0.253	−0.304 (−0.304)	−0.305 (−0.305)	−0.172	−0.445	3.7919
	B3PW91/TZVP	+0.342	−0.357 (−0.357)	−0.360 (−0.360)	−0.103	−0.384	1.6043
[Fe**L2**(O)F]	OPBE/TZVP	+0.212	−0.283 (−0.283)	−0.310 (−0.310)	−0.196	−0.458	3.7877
	B3PW91/TZVP	+0.433	−0.335 (−0.335)	−0.360 (−0.360)	−0.123	−0.489	3.7999
[Fe**L3**(O)F]	OPBE/TZVP	+0.103	−0.285 (−0.288)	−0.286 (−0.286)	−0.090	−0.386	1.1656
	B3PW91/TZVP	+0.212	−0.331 (−0.331)	−0.331 (−0.331)	−0.291	−0.545	3.8007

**Table 3:** Standard thermodynamic parameters of formation (enthalpy ∆*_f_H*^0^, entropy *S*^0^ and Gibbs’s energy ∆*_f_G*^0^) for the complexes having [Fe**L1**(O)F], [Fe**L2**(O)F] and [Fe**L3**(O)F] compositions calculated using DFT OPBE/TZVP method.

Complex	∆*H*^0^*_f_*_, 298_, kJ/mole	*S*^0^*_f_*_, 298_, J/mole K	∆*G*^0^*_f_*_, 298_, kJ/mole
[Fe**L1**(O)F]	200.1	772.2	421.0
[Fe**L2**(O)F]	143.7	955.6	387.7
[Fe**L3**(O)F]	85.3	1149.0	349.3

## Data Availability

No unpublished data was created or analyzed in this article.

## Supplementary Materials

The supporting information can be downloaded at: https://www.mdpi.com/article/10.3390/ijms24076442/s1.
