# Supplementary material for: Heteroligand Iron(V) Complexes Containing Porphyrazine, trans-Di[benzo]porphyrazine or Tetra[benzo]porphyrazine, Oxo and Fluoro Ligands: DFT Quantum-Chemical Study
Source: Int J Mol Sci. 2023 Mar 29;24(7):6442. doi: 10.3390/ijms24076442 (PMC10094394; doi:10.3390/ijms24076442)
Supplement: Supplementary file 1 [file ijms-24-06442-s001.zip › ijms-2276806-supplementary.pdf]

NBO Analysis Data for [FeL1(O)F]

B3PW91/TZVP

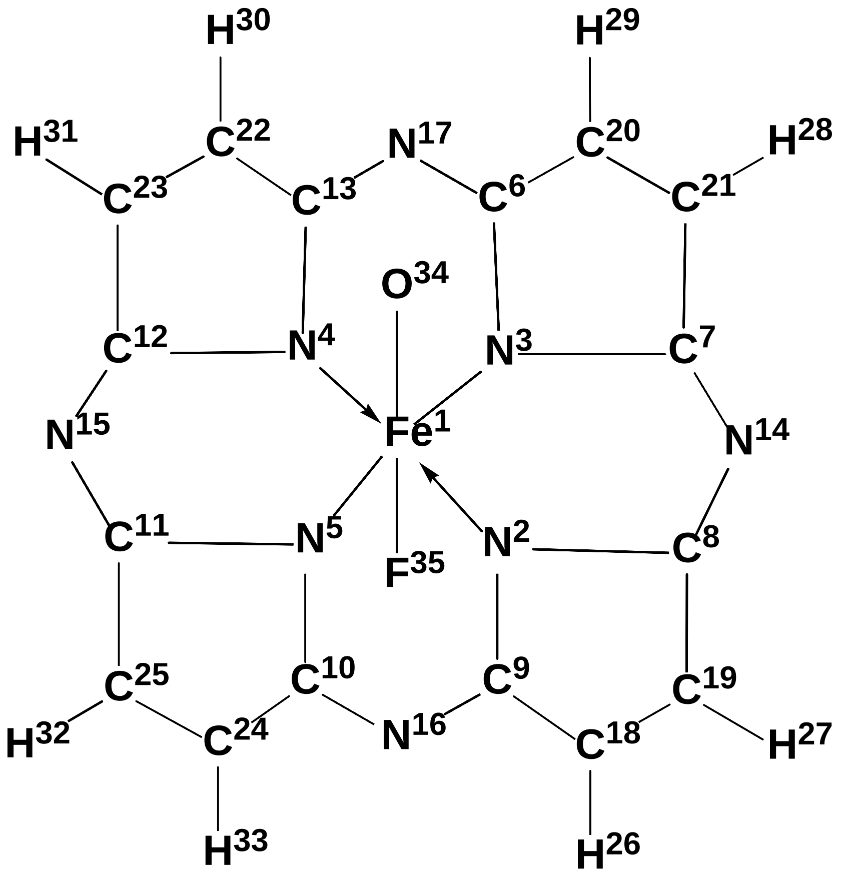

Dipole moment: = 0.8514 Debye

Mulliken charges and spin densities:

1	Fe	-0.135586	1.881296
2	N	0.135376	-0.073946
3	N	0.178366	-0.041873
4	N	0.135310	-0.074019
5	N	0.178376	-0.041874
6	C	0.005106	0.002257
7	C	0.005178	0.002259
8	C	0.012496	0.002807
9	C	0.012508	0.002810
10	C	0.005169	0.002263
11	C	0.005098	0.002260
12	C	0.012510	0.002807
13	C	0.012499	0.002804
14	N	-0.005322	-0.002124
15	N	-0.005305	-0.002119
16	N	-0.005314	-0.002126
17	N	-0.005313	-0.002117
18	C	-0.154987	0.002976
19	C	-0.154972	0.002979
20	C	-0.159968	0.002609
21	C	-0.159982	0.002609
22	C	-0.154977	0.002952
23	C	-0.154991	0.002949
24	C	-0.159979	0.002611
25	C	-0.159966	0.002611
26	H	0.140316	-0.000525
27	H	0.140316	-0.000525
28	H	0.139282	-0.000368
29	H	0.139282	-0.000368
30	H	0.140318	-0.000524
31	H	0.140317	-0.000524
32	H	0.139281	-0.000368
33	H	0.139281	-0.000368
34	O	-0.079946	-0.806256
35	F	-0.319781	0.126165

Sum of Mulliken charges = -0.00000 1.00000

ΔE(multipl.=2) = 0.0 кДж/моль
ΔE(multipl.=4) = 9.4 кДж/моль
ΔE(multipl.=6) = 94.1 кДж/моль
Alpha occupied eigenvalues (highest) = -6.424281 eV
Alpha virtual eigenvalues (lowest) = -4.4439372 eV
Beta occupied eigenvalues (highest) = -6.4093155 eV
Beta virtual eigenvalues (lowest) = -4.2398622 eV

<S**2>= 1.6043

Summary of Natural Population Analysis:

Atom	No	Natural Charge	Natural Population Core	Valence	Rydberg	Total
Fe	1	0.34191	17.99306	7.62378	0.04126	25.65809
N	2	-0.35698	1.99916	5.33057	0.02725	7.35698
N	3	-0.36010	1.99914	5.33375	0.02721	7.36010
N	4	-0.35698	1.99916	5.33057	0.02726	7.35698
N	5	-0.36009	1.99914	5.33375	0.02721	7.36009
C	6	0.35927	1.99927	3.61836	0.02311	5.64073
C	7	0.35926	1.99927	3.61837	0.02311	5.64074
C	8	0.36068	1.99926	3.61723	0.02283	5.63932
C	9	0.36067	1.99926	3.61724	0.02283	5.63933
C	10	0.35925	1.99927	3.61838	0.02311	5.64075
C	11	0.35926	1.99927	3.61837	0.02311	5.64074
C	12	0.36069	1.99926	3.61722	0.02283	5.63931
C	13	0.36070	1.99926	3.61721	0.02283	5.63930
N	14	-0.38962	1.99934	5.37463	0.01564	7.38962
N	15	-0.38961	1.99934	5.37463	0.01564	7.38961
N	16	-0.38961	1.99934	5.37462	0.01564	7.38961
N	17	-0.38962	1.99934	5.37464	0.01564	7.38962
C	18	-0.20838	1.99910	4.19588	0.01340	6.20838

C	19	-0.20837	1.99910	4.19587	0.01340	6.20837
C	20	-0.20644	1.99909	4.19399	0.01336	6.20644
C	21	-0.20644	1.99909	4.19399	0.01336	6.20644
C	22	-0.20838	1.99910	4.19588	0.01340	6.20838
C	23	-0.20839	1.99910	4.19589	0.01340	6.20839
C	24	-0.20645	1.99909	4.19400	0.01336	6.20645
C	25	-0.20645	1.99909	4.19400	0.01336	6.20645
H	26	0.23910	0.00000	0.75952	0.00138	0.76090
H	27	0.23910	0.00000	0.75952	0.00138	0.76090
H	28	0.24025	0.00000	0.75838	0.00137	0.75975
H	29	0.24025	0.00000	0.75838	0.00137	0.75975
H	30	0.23909	0.00000	0.75953	0.00138	0.76091
H	31	0.23910	0.00000	0.75952	0.00138	0.76090
H	32	0.24025	0.00000	0.75838	0.00137	0.75975
H	33	0.24025	0.00000	0.75838	0.00137	0.75975
O	34	-0.10336	1.99994	6.09693	0.00649	8.10336
F	35	-0.38377	1.99997	7.38018	0.00362	9.38377
* Total *		0.00000	69.97378	132.50155	0.52467	203.00000

NATURAL POPULATIONS: Natural atomic orbital occupancies

NAO	Atom	No	lang	Type(AO)	Occupancy
1	Fe	1	S	Cor(1S)	2.00000
2	Fe	1	S	Cor(2S)	1.99999
3	Fe	1	S	Cor(3S)	1.99650
4	Fe	1	S	Val(4S)	0.30244
5	Fe	1	S	Ryd(5S)	0.00153
6	Fe	1	S	Ryd(6S)	0.00051
7	Fe	1	px	Cor(2p)	2.00000
8	Fe	1	px	Cor(3p)	1.99862
9	Fe	1	px	Val(4p)	0.26348
10	Fe	1	px	Ryd(5p)	0.00076
11	Fe	1	py	Cor(2p)	2.00000
12	Fe	1	py	Cor(3p)	1.99860

13	Fe	1	py	Val(4p)	0.26705
14	Fe	1	py	Ryd(5p)	0.00075
15	Fe	1	pz	Cor(2p)	2.00000
16	Fe	1	pz	Cor(3p)	1.99935
17	Fe	1	pz	Val(4p)	0.24416
18	Fe	1	pz	Ryd(5p)	0.00270
19	Fe	1	dxy	Val(3d)	1.98779
20	Fe	1	dxy	Ryd(4d)	0.00431
21	Fe	1	dxy	Ryd(5d)	0.00055
22	Fe	1	dxz	Val(3d)	1.16337
23	Fe	1	dxz	Ryd(4d)	0.00620
24	Fe	1	dxz	Ryd(5d)	0.00011
25	Fe	1	dyz	Val(3d)	1.25689
26	Fe	1	dyz	Ryd(4d)	0.01064
27	Fe	1	dyz	Ryd(5d)	0.00013
28	Fe	1	dx2y2	Val(3d)	1.04341
29	Fe	1	dx2y2	Ryd(4d)	0.00523
30	Fe	1	dx2y2	Ryd(5d)	0.00005
31	Fe	1	dz2	Val(3d)	1.09519
32	Fe	1	dz2	Ryd(4d)	0.00765
33	Fe	1	dz2	Ryd(5d)	0.00014

NBO Analysis Data for [FeL1(O)F]

OPBE/TZVP

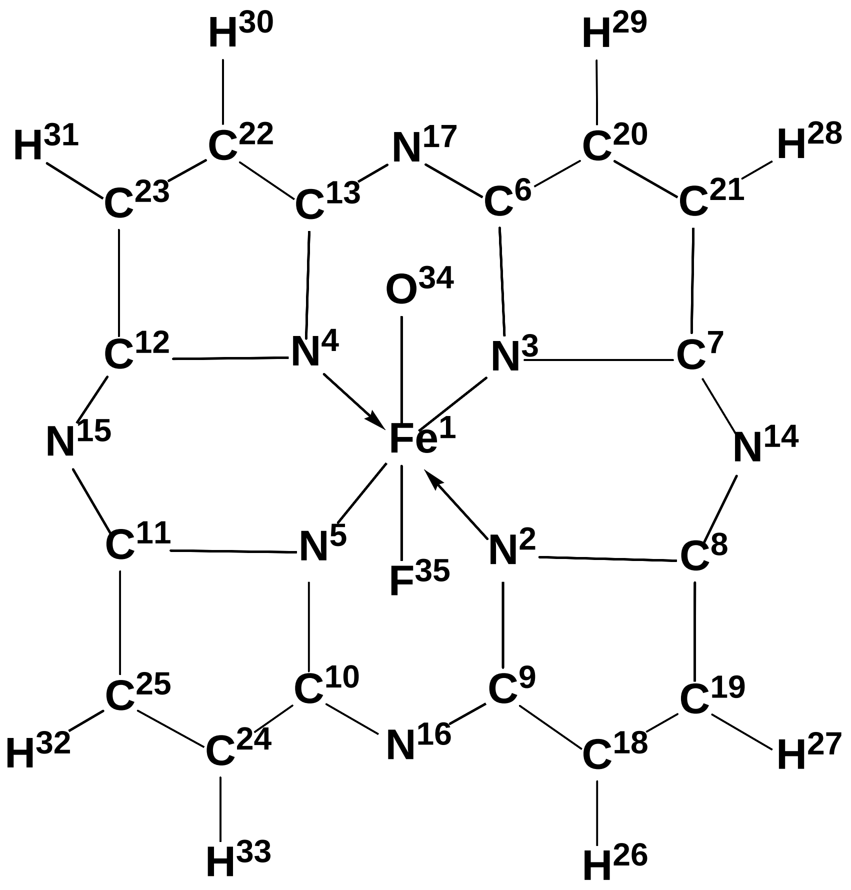

Dipole moment: = 0.7772 Debye

Mulliken charges and spin densities:

1	Fe	-0.380978	1.864880
2	N	0.410835	-0.032142
3	N	0.405062	-0.034112
4	N	0.410837	-0.032142
5	N	0.405063	-0.034112
6	C	-0.124963	0.013590
7	C	-0.124964	0.013590
8	C	-0.125640	0.013510
9	C	-0.125641	0.013510
10	C	-0.124962	0.013589
11	C	-0.124964	0.013590
12	C	-0.125643	0.013510
13	C	-0.125640	0.013510
14	N	0.282051	0.037224
15	N	0.282051	0.037224
16	N	0.282051	0.037224
17	N	0.282051	0.037224
18	C	-0.173836	0.000676
19	C	-0.173835	0.000675
20	C	-0.170369	0.000694
21	C	-0.170369	0.000693
22	C	-0.173837	0.000675
23	C	-0.173834	0.000676
24	C	-0.170369	0.000694
25	C	-0.170369	0.000693
26	H	0.057903	0.001259
27	H	0.057903	0.001259
28	H	0.057594	0.001052
29	H	0.057594	0.001052
30	H	0.057903	0.001259
31	H	0.057903	0.001259
32	H	0.057593	0.001052
33	H	0.057593	0.001052
34	O	-0.110264	0.952265
35	F	-0.351512	0.043349

Sum of Mulliken charges = -0.00000 3.00000

ΔE(multipl.=2) = 5.5 кДж/моль
ΔE(multipl.=4) = 0.0 кДж/моль
ΔE(multipl.=6) = 133.6 кДж/моль
Alpha occupied eigenvalues (highest) = -5.8945023 eV
Alpha virtual eigenvalues (lowest) = -4.0910235 eV
Beta occupied eigenvalues (highest) = -6.0177636 eV
Beta virtual eigenvalues (lowest) = -5.6338305 eV

<S**2>= 3.7919

Summary of Natural Population Analysis:

Atom	No	Natural Charge	Natural Population Core	Valence	Rydberg	Total
Fe	1	0.25285	17.99161	7.72080	0.03473	25.74715
N	2	-0.30431	1.99915	5.27794	0.02722	7.30431
N	3	-0.30513	1.99915	5.27875	0.02722	7.30513
N	4	-0.30431	1.99915	5.27793	0.02722	7.30431
N	5	-0.30513	1.99915	5.27875	0.02722	7.30513
C	6	0.33974	1.99927	3.64034	0.02065	5.66026
C	7	0.33974	1.99927	3.64034	0.02065	5.66026
C	8	0.33845	1.99927	3.64164	0.02064	5.66155
C	9	0.33845	1.99927	3.64164	0.02064	5.66155
C	10	0.33974	1.99927	3.64034	0.02065	5.66026
C	11	0.33974	1.99927	3.64034	0.02065	5.66026
C	12	0.33845	1.99927	3.64164	0.02064	5.66155
C	13	0.33845	1.99927	3.64164	0.02064	5.66155
N	14	-0.34012	1.99932	5.32627	0.01452	7.34012
N	15	-0.34012	1.99932	5.32627	0.01452	7.34012
N	16	-0.34012	1.99932	5.32627	0.01452	7.34012
N	17	-0.34012	1.99932	5.32627	0.01452	7.34012
C	18	-0.21701	1.99910	4.20633	0.01158	6.21701
C	19	-0.21701	1.99910	4.20633	0.01158	6.21701
C	20	-0.21695	1.99910	4.20628	0.01156	6.21695

C	21	-0.21695	1.99910	4.20628	0.01156	6.21695
C	22	-0.21701	1.99910	4.20633	0.01158	6.21701
C	23	-0.21701	1.99910	4.20633	0.01158	6.21701
C	24	-0.21695	1.99910	4.20628	0.01156	6.21695
C	25	-0.21695	1.99910	4.20628	0.01156	6.21695
H	26	0.24577	0.00000	0.75269	0.00154	0.75423
H	27	0.24577	0.00000	0.75269	0.00154	0.75423
H	28	0.24579	0.00000	0.75266	0.00154	0.75421
H	29	0.24579	0.00000	0.75266	0.00154	0.75421
H	30	0.24577	0.00000	0.75269	0.00154	0.75423
H	31	0.24577	0.00000	0.75269	0.00154	0.75423
H	32	0.24579	0.00000	0.75266	0.00154	0.75421
H	33	0.24579	0.00000	0.75266	0.00154	0.75421
O	34	-0.17201	1.99994	6.16579	0.00628	8.17201
F	35	-0.44469	1.99998	7.44207	0.00264	9.44469
* Total *		-0.00000	69.97242	132.54691	0.48067	203.00000

NATURAL POPULATIONS: Natural atomic orbital occupancies

NAO	Atom	No	lang	Type(AO)	Occupancy
1	Fe	1	S	Cor(1S)	2.00000
2	Fe	1	S	Cor(2S)	2.00000
3	Fe	1	S	Cor(3S)	1.99627
4	Fe	1	S	Val(4S)	0.29836
5	Fe	1	S	Ryd(5S)	0.00187
6	Fe	1	S	Ryd(6S)	0.00049
7	Fe	1	px	Cor(2p)	2.00000
8	Fe	1	px	Cor(3p)	1.99849
9	Fe	1	px	Val(4p)	0.25947
10	Fe	1	px	Ryd(5p)	0.00082
11	Fe	1	py	Cor(2p)	2.00000
12	Fe	1	py	Cor(3p)	1.99847
13	Fe	1	py	Val(4p)	0.25875
14	Fe	1	py	Ryd(5p)	0.00076
15	Fe	1	pz	Cor(2p)	2.00000

16	Fe	1	pz	Cor(3p)	1.99839
17	Fe	1	pz	Val(4p)	0.23918
18	Fe	1	pz	Ryd(5p)	0.00216
19	Fe	1	dxy	Val(3d)	1.33806
20	Fe	1	dxy	Ryd(4d)	0.00359
21	Fe	1	dxy	Ryd(5d)	0.00050
22	Fe	1	dxz	Val(3d)	1.52684
23	Fe	1	dxz	Ryd(4d)	0.00695
24	Fe	1	dxz	Ryd(5d)	0.00014
25	Fe	1	dyz	Val(3d)	1.53245
26	Fe	1	dyz	Ryd(4d)	0.00672
27	Fe	1	dyz	Ryd(5d)	0.00014
28	Fe	1	dx2y2	Val(3d)	1.12878
29	Fe	1	dx2y2	Ryd(4d)	0.00406
30	Fe	1	dx2y2	Ryd(5d)	0.00005
31	Fe	1	dz2	Val(3d)	1.13893
32	Fe	1	dz2	Ryd(4d)	0.00641
33	Fe	1	dz2	Ryd(5d)	0.00006

NBO Analysis Data for [FeL2(O)F]

B3PW91/TZVP

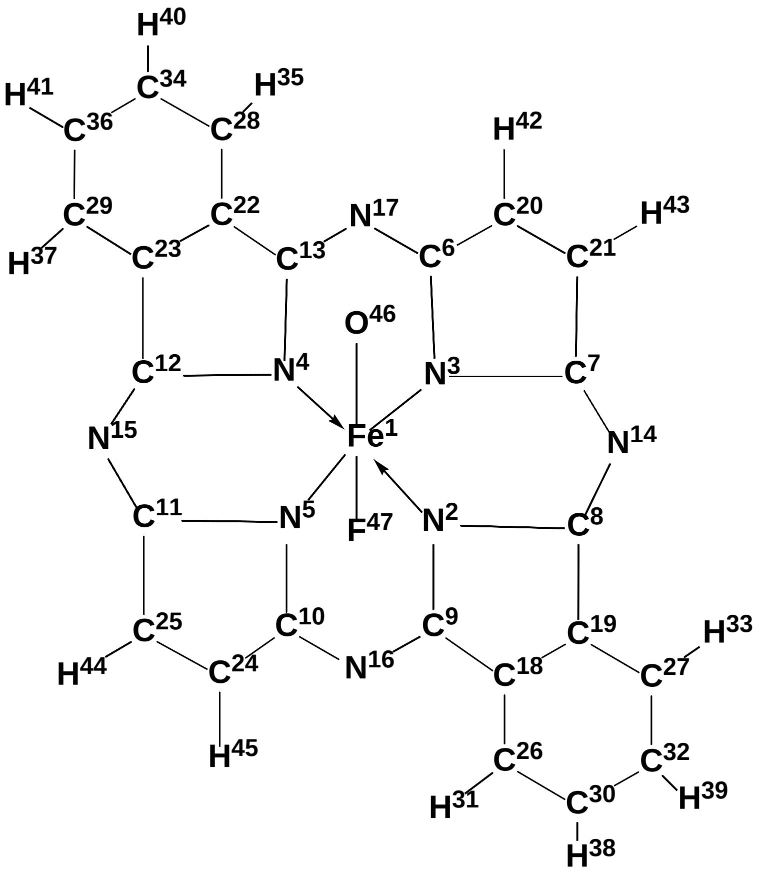

Dipole moment: = 0.4184 Debye

Mulliken charges and spin densities:

1	Fe	0.040388	1.196631
2	N	0.175681	-0.075659
3	N	0.174797	-0.073794
4	N	0.175693	-0.075663
5	N	0.174804	-0.073800
6	C	0.025642	0.163818
7	C	0.025638	0.163811
8	C	-0.018220	0.161780
9	C	-0.018215	0.161788
10	C	0.025644	0.163825
11	C	0.025638	0.163833
12	C	-0.018219	0.161798
13	C	-0.018215	0.161789
14	N	-0.027912	-0.072124
15	N	-0.027908	-0.072132
16	N	-0.027910	-0.072128
17	N	-0.027911	-0.072127
18	C	0.038839	-0.029557
19	C	0.038841	-0.029553
20	C	-0.184366	0.016877
21	C	-0.184358	0.016886
22	C	0.038837	-0.029555
23	C	0.038843	-0.029559
24	C	-0.184365	0.016889
25	C	-0.184361	0.016878
26	C	-0.146192	0.047272
27	C	-0.146189	0.047268
28	C	-0.146189	0.047271
29	C	-0.146191	0.047275
30	C	-0.071713	0.012948
31	H	0.126822	-0.001632
32	C	-0.071714	0.012953
33	H	0.126822	-0.001632
34	C	-0.071714	0.012954
35	H	0.126822	-0.001632
36	C	-0.071713	0.012949
37	H	0.126822	-0.001632
38	H	0.121022	-0.001154
39	H	0.121022	-0.001155
40	H	0.121022	-0.001155
41	H	0.121022	-0.001155
42	H	0.138844	-0.001534
43	H	0.138844	-0.001535
44	H	0.138844	-0.001535
45	H	0.138844	-0.001535
46	O	-0.278840	0.891349
47	F	-0.473623	0.024096

Sum of Mulliken charges = -0.00000 3.00000

ΔE(multipl.=2) = 49.9 кДж/моль
ΔE(multipl.=4) = 0.0 кДж/моль
ΔE(multipl.=6) = 77.2 кДж/моль
Alpha occupied eigenvalues (highest) = -6.1913634 eV
Alpha virtual eigenvalues (lowest) = -3.6292698 eV
Beta occupied eigenvalues (highest) = -6.4299951 eV
Beta virtual eigenvalues (lowest) = -4.9524921 eV

<S**2>= 3.7999

Summary of Natural Population Analysis:

			Natural Population			
Atom	No	Natural Charge	Core	Valence	Rydberg	Total
Fe	1	0.43293	17.99234	7.53464	0.04010	25.56707
N	2	-0.33454	1.99914	5.30764	0.02777	7.33454
N	3	-0.36034	1.99909	5.33376	0.02749	7.36034
N	4	-0.33454	1.99914	5.30763	0.02777	7.33454
N	5	-0.36034	1.99909	5.33376	0.02749	7.36034
C	6	0.36252	1.99929	3.61495	0.02325	5.63748
C	7	0.36252	1.99929	3.61495	0.02325	5.63748
C	8	0.39038	1.99921	3.58807	0.02234	5.60962
C	9	0.39038	1.99921	3.58807	0.02234	5.60962
C	10	0.36251	1.99929	3.61495	0.02325	5.63749
C	11	0.36251	1.99929	3.61495	0.02325	5.63749
C	12	0.39038	1.99921	3.58807	0.02234	5.60962
C	13	0.39038	1.99921	3.58807	0.02234	5.60962
N	14	-0.39926	1.99934	5.38473	0.01519	7.39926
N	15	-0.39925	1.99934	5.38472	0.01519	7.39925
N	16	-0.39926	1.99934	5.38473	0.01519	7.39926
N	17	-0.39926	1.99934	5.38473	0.01519	7.39926
C	18	-0.07459	1.99901	4.05839	0.01719	6.07459

C	19	-0.07459	1.99901	4.05839	0.01719	6.07459
C	20	-0.21322	1.99907	4.20082	0.01333	6.21322
C	21	-0.21322	1.99907	4.20082	0.01333	6.21322
C	22	-0.07459	1.99901	4.05839	0.01719	6.07459
C	23	-0.07459	1.99901	4.05839	0.01719	6.07459
C	24	-0.21322	1.99907	4.20083	0.01333	6.21322
C	25	-0.21322	1.99907	4.20083	0.01333	6.21322
C	26	-0.16687	1.99911	4.15409	0.01366	6.16687
C	27	-0.16687	1.99911	4.15409	0.01366	6.16687
C	28	-0.16687	1.99911	4.15409	0.01366	6.16687
C	29	-0.16687	1.99911	4.15409	0.01366	6.16687
C	30	-0.19900	1.99925	4.18582	0.01393	6.19900
H	31	0.23390	0.00000	0.76447	0.00163	0.76610
C	32	-0.19900	1.99925	4.18582	0.01393	6.19900
H	33	0.23390	0.00000	0.76447	0.00163	0.76610
C	34	-0.19900	1.99925	4.18582	0.01393	6.19900
H	35	0.23390	0.00000	0.76447	0.00163	0.76610
C	36	-0.19900	1.99925	4.18582	0.01393	6.19900
H	37	0.23390	0.00000	0.76447	0.00163	0.76610
H	38	0.21924	0.00000	0.77965	0.00111	0.78076
H	39	0.21924	0.00000	0.77965	0.00111	0.78076
H	40	0.21924	0.00000	0.77965	0.00111	0.78076
H	41	0.21924	0.00000	0.77965	0.00111	0.78076
H	42	0.23915	0.00000	0.75946	0.00139	0.76085
H	43	0.23915	0.00000	0.75946	0.00139	0.76085
H	44	0.23915	0.00000	0.75946	0.00139	0.76085
H	45	0.23915	0.00000	0.75946	0.00139	0.76085
O	46	-0.12349	1.99993	6.11658	0.00698	8.12349
F	47	-0.48870	1.99998	7.48604	0.00268	9.48870
* Total *		0.00000	85.96584	168.38181	0.65236	255.00000

NATURAL POPULATIONS: Natural atomic orbital occupancies

NAO	Atom	No	lang	Type(AO)	Occupancy

1	Fe	1	S	Cor(1S)	2.00000
2	Fe	1	S	Cor(2S)	1.99999
3	Fe	1	S	Cor(3S)	1.99657
4	Fe	1	S	Val(4S)	0.30115
5	Fe	1	S	Ryd(5S)	0.00131
6	Fe	1	S	Ryd(6S)	0.00057
7	Fe	1	px	Cor(2p)	2.00000
8	Fe	1	px	Cor(3p)	1.99869
9	Fe	1	px	Val(4p)	0.26332
10	Fe	1	px	Ryd(5p)	0.00053
11	Fe	1	py	Cor(2p)	2.00000
12	Fe	1	py	Cor(3p)	1.99869
13	Fe	1	py	Val(4p)	0.26632
14	Fe	1	py	Ryd(5p)	0.00079
15	Fe	1	pz	Cor(2p)	2.00000
16	Fe	1	pz	Cor(3p)	1.99840
17	Fe	1	pz	Val(4p)	0.24333
18	Fe	1	pz	Ryd(5p)	0.00235
19	Fe	1	dxy	Val(3d)	1.07480
20	Fe	1	dxy	Ryd(4d)	0.00475
21	Fe	1	dxy	Ryd(5d)	0.00071
22	Fe	1	dxz	Val(3d)	1.58792
23	Fe	1	dxz	Ryd(4d)	0.00790
24	Fe	1	dxz	Ryd(5d)	0.00014
25	Fe	1	dyz	Val(3d)	1.58080
26	Fe	1	dyz	Ryd(4d)	0.00793
27	Fe	1	dyz	Ryd(5d)	0.00014
28	Fe	1	dx2y2	Val(3d)	1.14019
29	Fe	1	dx2y2	Ryd(4d)	0.00520
30	Fe	1	dx2y2	Ryd(5d)	0.00004
31	Fe	1	dz2	Val(3d)	1.07681
32	Fe	1	dz2	Ryd(4d)	0.00766
33	Fe	1	dz2	Ryd(5d)	0.00010

NBO Analysis Data for [FeL2(O)F]

OPBE/TZVP

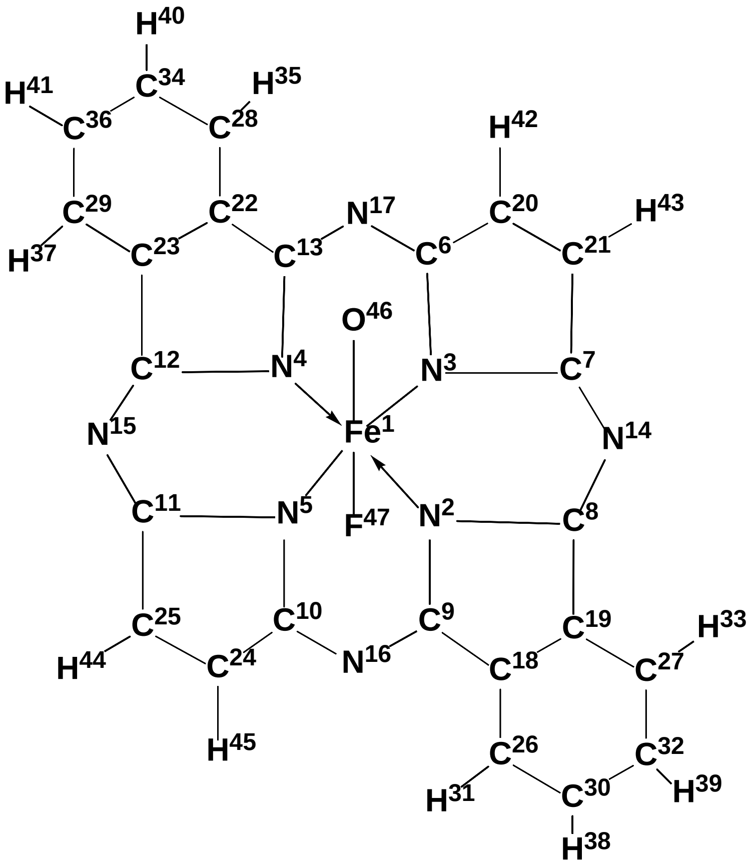

Dipole moment: = 0.3468 Debye

Mulliken charges and spin densities:

1	Fe	-0.332938	1.686941
2	N	0.419547	-0.045321
3	N	0.410080	-0.041986
4	N	0.419560	-0.045321
5	N	0.410094	-0.041987
6	C	-0.112933	0.047752
7	C	-0.112949	0.047754
8	C	-0.206623	0.046298
9	C	-0.206629	0.046299
10	C	-0.112929	0.047754
11	C	-0.112948	0.047751
12	C	-0.206618	0.046296
13	C	-0.206623	0.046296
14	N	0.268475	0.014361
15	N	0.268475	0.014361
16	N	0.268474	0.014360
17	N	0.268475	0.014361
18	C	0.228716	-0.010687
19	C	0.228712	-0.010688
20	C	-0.207943	0.007081
21	C	-0.207921	0.007075
22	C	0.228712	-0.010688
23	C	0.228712	-0.010687
24	C	-0.207945	0.007077
25	C	-0.207927	0.007079
26	C	-0.341156	0.018415
27	C	-0.341154	0.018415
28	C	-0.341156	0.018415
29	C	-0.341159	0.018414
30	C	-0.012215	0.004527
31	H	0.062566	-0.000687
32	C	-0.012217	0.004527
33	H	0.062567	-0.000687
34	C	-0.012217	0.004526
35	H	0.062566	-0.000687
36	C	-0.012215	0.004527
37	H	0.062566	-0.000687
38	H	0.062599	-0.000498
39	H	0.062599	-0.000498
40	H	0.062599	-0.000498
41	H	0.062599	-0.000498
42	H	0.053497	0.000159
43	H	0.053497	0.000159
44	H	0.053497	0.000159
45	H	0.053497	0.000159
46	O	-0.137996	0.935075
47	F	-0.368272	0.045732

Sum of Mulliken charges = -0.00000 3.00000

ΔE(multipl.=2) = 2.7 кДж/моль
ΔE(multipl.=4) = 0.0 кДж/моль
ΔE(multipl.=6) = 107.1 кДж/моль
Alpha occupied eigenvalues (highest) = -5.4248577 eV
Alpha virtual eigenvalues (lowest) = -3.9816393 eV
Beta occupied eigenvalues (highest) = -5.537235 eV
Beta virtual eigenvalues (lowest) = -5.2322109 eV

<S**2>= 3.7877

Summary of Natural Population Analysis:

			Natural Population			
Atom	No	Natural Charge	Core	Valence	Rydberg	Total
Fe	1	0.21168	17.99147	7.76335	0.03350	25.78832
N	2	-0.28273	1.99919	5.25640	0.02714	7.28273
N	3	-0.31000	1.99916	5.28422	0.02663	7.31000
N	4	-0.28273	1.99919	5.25640	0.02714	7.28273
N	5	-0.31000	1.99916	5.28421	0.02663	7.31000
C	6	0.35100	1.99928	3.62888	0.02084	5.64900
C	7	0.35100	1.99928	3.62888	0.02084	5.64900
C	8	0.38355	1.99921	3.59701	0.02024	5.61645
C	9	0.38355	1.99921	3.59701	0.02024	5.61645
C	10	0.35100	1.99928	3.62888	0.02084	5.64900
C	11	0.35100	1.99928	3.62888	0.02084	5.64900
C	12	0.38355	1.99921	3.59701	0.02024	5.61645
C	13	0.38355	1.99921	3.59701	0.02024	5.61645
N	14	-0.36513	1.99933	5.35117	0.01464	7.36513
N	15	-0.36513	1.99933	5.35117	0.01464	7.36513
N	16	-0.36513	1.99933	5.35117	0.01464	7.36513
N	17	-0.36513	1.99933	5.35117	0.01464	7.36513
C	18	-0.07927	1.99904	4.06465	0.01558	6.07927
C	19	-0.07927	1.99904	4.06465	0.01558	6.07927
C	20	-0.21995	1.99910	4.20922	0.01163	6.21995

C	21	-0.21995	1.99910	4.20922	0.01163	6.21995
C	22	-0.07927	1.99904	4.06465	0.01558	6.07927
C	23	-0.07927	1.99904	4.06465	0.01558	6.07927
C	24	-0.21995	1.99910	4.20922	0.01163	6.21995
C	25	-0.21995	1.99910	4.20922	0.01163	6.21995
C	26	-0.16699	1.99913	4.15608	0.01177	6.16699
C	27	-0.16699	1.99913	4.15608	0.01177	6.16699
C	28	-0.16699	1.99913	4.15608	0.01177	6.16699
C	29	-0.16699	1.99913	4.15608	0.01177	6.16699
C	30	-0.20371	1.99926	4.19272	0.01173	6.20371
H	31	0.23900	0.00000	0.75918	0.00182	0.76100
C	32	-0.20371	1.99926	4.19272	0.01173	6.20371
H	33	0.23900	0.00000	0.75918	0.00182	0.76100
C	34	-0.20371	1.99926	4.19272	0.01173	6.20371
H	35	0.23900	0.00000	0.75918	0.00182	0.76100
C	36	-0.20371	1.99926	4.19272	0.01173	6.20371
H	37	0.23900	0.00000	0.75918	0.00182	0.76100
H	38	0.22547	0.00000	0.77330	0.00123	0.77453
H	39	0.22547	0.00000	0.77330	0.00123	0.77453
H	40	0.22547	0.00000	0.77330	0.00123	0.77453
H	41	0.22547	0.00000	0.77330	0.00123	0.77453
H	42	0.24312	0.00000	0.75529	0.00159	0.75688
H	43	0.24312	0.00000	0.75529	0.00159	0.75688
H	44	0.24312	0.00000	0.75529	0.00159	0.75688
H	45	0.24312	0.00000	0.75529	0.00159	0.75688
O	46	-0.19641	1.99994	6.19037	0.00611	8.19641
F	47	-0.45819	1.99998	7.45582	0.00239	9.45819
* Total *		0.00000	85.96544	168.44076	0.59380	255.00000

NATURAL POPULATIONS: Natural atomic orbital occupancies

NAO	Atom	No	lang	Type(AO)	Occupancy
1	Fe	1	S	Cor(1S)	2.00000
2	Fe	1	S	Cor(2S)	2.00000

3	Fe	1	S	Cor(3S)	1.99622
4	Fe	1	S	Val(4S)	0.29335
5	Fe	1	S	Ryd(5S)	0.00155
6	Fe	1	S	Ryd(6S)	0.00057
7	Fe	1	px	Cor(2p)	2.00000
8	Fe	1	px	Cor(3p)	1.99845
9	Fe	1	px	Val(4p)	0.25260
10	Fe	1	px	Ryd(5p)	0.00053
11	Fe	1	py	Cor(2p)	2.00000
12	Fe	1	py	Cor(3p)	1.99848
13	Fe	1	py	Val(4p)	0.25649
14	Fe	1	py	Ryd(5p)	0.00083
15	Fe	1	pz	Cor(2p)	2.00000
16	Fe	1	pz	Cor(3p)	1.99834
17	Fe	1	pz	Val(4p)	0.23539
18	Fe	1	pz	Ryd(5p)	0.00213
19	Fe	1	dxy	Val(3d)	1.51059
20	Fe	1	dxy	Ryd(4d)	0.00362
21	Fe	1	dxy	Ryd(5d)	0.00052
22	Fe	1	dxz	Val(3d)	1.51294
23	Fe	1	dxz	Ryd(4d)	0.00664
24	Fe	1	dxz	Ryd(5d)	0.00014
25	Fe	1	dyz	Val(3d)	1.50860
26	Fe	1	dyz	Ryd(4d)	0.00696
27	Fe	1	dyz	Ryd(5d)	0.00015
28	Fe	1	dx2y2	Val(3d)	1.07396
29	Fe	1	dx2y2	Ryd(4d)	0.00358
30	Fe	1	dx2y2	Ryd(5d)	0.00006
31	Fe	1	dz2	Val(3d)	1.11942
32	Fe	1	dz2	Ryd(4d)	0.00615
33	Fe	1	dz2	Ryd(5d)	0.00007

NBO Analysis Data for [FeL3(O)F]

B3PW91/TZVP

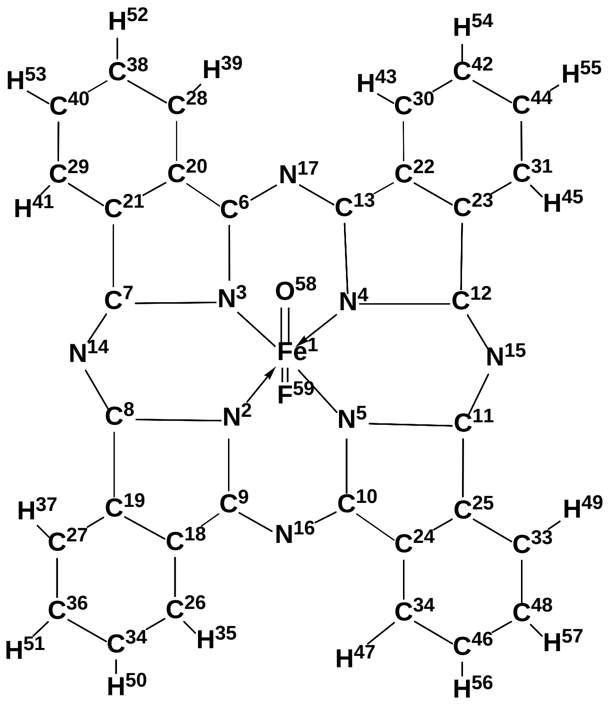

Dipole moment: = 0.4198 Debye

Mulliken charges and spin densities:

1	Fe	0.052951	1.198107
2	N	0.180677	-0.072814
3	N	0.180670	-0.072814
4	N	0.180676	-0.072814
5	N	0.180669	-0.072815
6	C	0.016555	0.156946
7	C	0.016553	0.156945
8	C	0.016556	0.156944
9	C	0.016557	0.156944
10	C	0.016554	0.156946
11	C	0.016554	0.156946
12	C	0.016557	0.156945
13	C	0.016555	0.156945
14	N	-0.040381	-0.067533
15	N	-0.040381	-0.067533
16	N	-0.040381	-0.067533
17	N	-0.040381	-0.067533
18	C	-0.022649	-0.025528
19	C	-0.022648	-0.025527
20	C	-0.022647	-0.025527
21	C	-0.022647	-0.025528
22	C	-0.022648	-0.025528
23	C	-0.022648	-0.025528
24	C	-0.022646	-0.025527
25	C	-0.022647	-0.025528
26	C	-0.146129	0.039236
27	C	-0.146130	0.039236
28	C	-0.146131	0.039235
29	C	-0.146130	0.039235
30	C	-0.146129	0.039236
31	C	-0.146130	0.039236
32	C	-0.146131	0.039235
33	C	-0.146130	0.039236
34	C	-0.072620	0.012694
35	H	0.125103	-0.001256
36	C	-0.072619	0.012694
37	H	0.125103	-0.001256
38	C	-0.072619	0.012694
39	H	0.125103	-0.001256
40	C	-0.072619	0.012694
41	H	0.125103	-0.001256
42	C	-0.072620	0.012694
43	H	0.125104	-0.001256
44	C	-0.072619	0.012694
45	H	0.125103	-0.001256
46	C	-0.072619	0.012694
47	H	0.125103	-0.001256
48	C	-0.072619	0.012694
49	H	0.125103	-0.001256
50	H	0.116895	-0.001131
51	H	0.116896	-0.001131
52	H	0.116896	-0.001131
53	H	0.116896	-0.001131
54	H	0.116896	-0.001131
55	H	0.116895	-0.001131
56	H	0.116896	-0.001131
57	H	0.116896	-0.001131
58	O	-0.279147	0.889872
59	F	-0.472231	0.025726

Sum of Mulliken charges = -0.00000 3.00000

ΔE(multipl.=2) = 59.0 кДж/моль
ΔE(multipl.=4) = 0.0 кДж/моль
ΔE(multipl.=6) = 183.8 кДж/моль
Alpha occupied eigenvalues (highest) = -5.7497451 eV
Alpha virtual eigenvalues (lowest) = -3.2752677 eV
Beta occupied eigenvalues (highest) = -6.3848265 eV
Beta virtual eigenvalues (lowest) = -4.5993063 eV

<S**2>= 3.8007

Summary of Natural Population Analysis:

			Natural Population			
Atom	No	Natural Charge	Core	Valence	Rydberg	Total
Fe	1	0.21202	17.99133	7.75922	0.03743	25.78798
N	2	-0.33099	1.99919	5.30421	0.02759	7.33099
N	3	-0.33099	1.99919	5.30421	0.02759	7.33099
N	4	-0.33099	1.99919	5.30421	0.02759	7.33099
N	5	-0.33100	1.99919	5.30421	0.02759	7.33100
C	6	0.44916	1.99922	3.52881	0.02282	5.55084
C	7	0.44916	1.99922	3.52881	0.02282	5.55084
C	8	0.44916	1.99922	3.52881	0.02282	5.55084
C	9	0.44916	1.99922	3.52881	0.02282	5.55084
C	10	0.44916	1.99922	3.52881	0.02282	5.55084
C	11	0.44916	1.99922	3.52881	0.02282	5.55084
C	12	0.44916	1.99922	3.52881	0.02282	5.55084
C	13	0.44916	1.99922	3.52881	0.02282	5.55084
N	14	-0.44054	1.99933	5.42510	0.01611	7.44054
N	15	-0.44054	1.99933	5.42510	0.01611	7.44054
N	16	-0.44054	1.99933	5.42510	0.01611	7.44054
N	17	-0.44054	1.99933	5.42510	0.01611	7.44054
C	18	-0.08240	1.99901	4.06617	0.01721	6.08240

C	19	-0.08239	1.99901	4.06617	0.01721	6.08239
C	20	-0.08239	1.99901	4.06617	0.01721	6.08239
C	21	-0.08239	1.99901	4.06617	0.01721	6.08239
C	22	-0.08240	1.99901	4.06617	0.01721	6.08240
C	23	-0.08239	1.99901	4.06617	0.01721	6.08239
C	24	-0.08239	1.99901	4.06617	0.01721	6.08239
C	25	-0.08240	1.99901	4.06617	0.01721	6.08240
C	26	-0.15777	1.99912	4.14500	0.01366	6.15777
C	27	-0.15777	1.99912	4.14500	0.01366	6.15777
C	28	-0.15777	1.99912	4.14500	0.01366	6.15777
C	29	-0.15777	1.99912	4.14500	0.01366	6.15777
C	30	-0.15777	1.99912	4.14500	0.01366	6.15777
C	31	-0.15777	1.99912	4.14500	0.01366	6.15777
C	32	-0.15777	1.99912	4.14500	0.01366	6.15777
C	33	-0.15777	1.99912	4.14500	0.01366	6.15777
C	34	-0.19709	1.99925	4.18397	0.01387	6.19709
H	35	0.23287	0.00000	0.76546	0.00167	0.76713
C	36	-0.19709	1.99925	4.18397	0.01387	6.19709
H	37	0.23287	0.00000	0.76546	0.00167	0.76713
C	38	-0.19709	1.99925	4.18397	0.01387	6.19709
H	39	0.23287	0.00000	0.76546	0.00167	0.76713
C	40	-0.19709	1.99925	4.18397	0.01387	6.19709
H	41	0.23287	0.00000	0.76546	0.00167	0.76713
C	42	-0.19709	1.99925	4.18397	0.01387	6.19709
H	43	0.23287	0.00000	0.76546	0.00167	0.76713
C	44	-0.19709	1.99925	4.18397	0.01387	6.19709
H	45	0.23287	0.00000	0.76546	0.00167	0.76713
C	46	-0.19709	1.99925	4.18397	0.01387	6.19709
H	47	0.23287	0.00000	0.76546	0.00167	0.76713
C	48	-0.19709	1.99925	4.18397	0.01387	6.19709
H	49	0.23287	0.00000	0.76546	0.00167	0.76713
H	50	0.21902	0.00000	0.77986	0.00112	0.78098
H	51	0.21902	0.00000	0.77986	0.00112	0.78098
H	52	0.21902	0.00000	0.77986	0.00112	0.78098
H	53	0.21902	0.00000	0.77986	0.00112	0.78098
H	54	0.21902	0.00000	0.77986	0.00112	0.78098
H	55	0.21902	0.00000	0.77986	0.00112	0.78098

H	56	0.21902	0.00000	0.77986	0.00112	0.78098
H	57	0.21902	0.00000	0.77986	0.00112	0.78098
O	58	-0.29117	1.99993	6.28485	0.00639	8.29117
F	59	-0.54506	1.99998	7.54282	0.00226	9.54506
* Total *		0.00000	101.95811	204.25817	0.78372	307.00000

NATURAL POPULATIONS: Natural atomic orbital occupancies

NAO	Atom	No	lang	Type(AO)	Occupancy
1	Fe	1	S	Cor(1S)	2.00000
2	Fe	1	S	Cor(2S)	2.00000
3	Fe	1	S	Cor(3S)	1.99626
4	Fe	1	S	Val(4S)	0.27834
5	Fe	1	S	Ryd(5S)	0.00105
6	Fe	1	S	Ryd(6S)	0.00050
7	Fe	1	px	Cor(2p)	2.00000
8	Fe	1	px	Cor(3p)	1.99856
9	Fe	1	px	Val(4p)	0.25037
10	Fe	1	px	Ryd(5p)	0.00040
11	Fe	1	py	Cor(2p)	2.00000
12	Fe	1	py	Cor(3p)	1.99856
13	Fe	1	py	Val(4p)	0.25037
14	Fe	1	py	Ryd(5p)	0.00040
15	Fe	1	pz	Cor(2p)	2.00000
16	Fe	1	pz	Cor(3p)	1.99796
17	Fe	1	pz	Val(4p)	0.23233
18	Fe	1	pz	Ryd(5p)	0.00207
19	Fe	1	dxy	Val(3d)	1.98761
20	Fe	1	dxy	Ryd(4d)	0.00453
21	Fe	1	dxy	Ryd(5d)	0.00059
22	Fe	1	dxz	Val(3d)	1.46458
23	Fe	1	dxz	Ryd(4d)	0.00820
24	Fe	1	dxz	Ryd(5d)	0.00013
25	Fe	1	dyz	Val(3d)	1.46458

26	Fe	1	dyz	Ryd(4d)	0.00820
27	Fe	1	dyz	Ryd(5d)	0.00013
28	Fe	1	dx2y2	Val(3d)	0.83362
29	Fe	1	dx2y2	Ryd(4d)	0.00378
30	Fe	1	dx2y2	Ryd(5d)	0.00005
31	Fe	1	dz2	Val(3d)	0.99741
32	Fe	1	dz2	Ryd(4d)	0.00729
33	Fe	1	dz2	Ryd(5d)	0.00011

NBO Analysis Data for [FeL3(O)F]

OPBE/TZVP

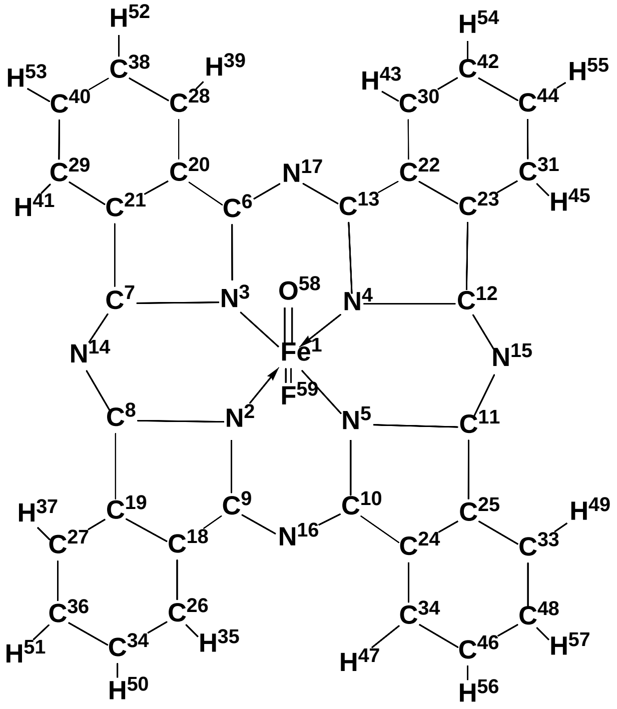

Dipole moment: = 0.6717 Debye

Mulliken charges and spin densities:

1	Fe	-0.318788	0.996387
2	N	0.443238	0.003997
3	N	0.431341	0.004996
4	N	0.442177	0.014215
5	N	0.431336	0.004959
6	C	-0.128201	-0.103868
7	C	-0.145149	-0.068883
8	C	-0.141831	-0.070804
9	C	-0.142012	-0.070700
10	C	-0.144966	-0.068779
11	C	-0.128347	-0.103780
12	C	-0.143763	-0.102094
13	C	-0.143628	-0.102193
14	N	0.255303	0.022811
15	N	0.254912	0.030698
16	N	0.255297	0.022787
17	N	0.254911	0.030721
18	C	0.144518	0.012120
19	C	0.144431	0.012174
20	C	0.136028	0.022723
21	C	0.146367	0.005594
22	C	0.145208	0.016995
23	C	0.145349	0.016932
24	C	0.146230	0.005574
25	C	0.136076	0.022717
26	C	-0.357196	-0.024153
27	C	-0.357247	-0.024190
28	C	-0.352264	-0.035181
29	C	-0.352961	-0.022116
30	C	-0.354793	-0.033555
31	C	-0.354800	-0.033518
32	C	-0.352970	-0.022083
33	C	-0.352175	-0.035158
34	C	-0.008296	-0.007122
35	H	0.054601	0.001042
36	C	-0.008275	-0.007074
37	H	0.054593	0.001045
38	C	-0.009470	-0.000655
39	H	0.055499	0.001632
40	C	-0.007704	-0.017342
41	H	0.056251	0.000793
42	C	-0.008152	-0.010593
43	H	0.056009	0.001406
44	C	-0.008145	-0.010642
45	H	0.056014	0.001404
46	C	-0.007691	-0.017339
47	H	0.056246	0.000791
48	C	-0.009498	-0.000638
49	H	0.055509	0.001631
50	H	0.057439	0.000701
51	H	0.057438	0.000698
52	H	0.058049	0.000356
53	H	0.058271	0.001404
54	H	0.058605	0.001051
55	H	0.058606	0.001054
56	H	0.058267	0.001404
57	H	0.058047	0.000355
58	O	-0.133317	0.701514
59	F	-0.350527	0.027777

Sum of Mulliken charges = -0.00000 1.00000

ΔE(multipl.=2) = 0.0 кДж/моль
ΔE(multipl.=4) = 1.1 кДж/моль
ΔE(multipl.=6) = 134.1 кДж/моль
Alpha occupied eigenvalues (highest) = -4.9927629 eV
Alpha virtual eigenvalues (lowest) = -4.9905861 eV
Beta occupied eigenvalues (highest) = -5.1737094 eV
Beta virtual eigenvalues (lowest) = -3.7136208 eV

<S**2>= 1.1656

Summary of Natural Population Analysis:

		Natural	Natural Population			
Atom	No	Charge	Core	Valence	Rydberg	Total
Fe	1	0.10341	17.99170	7.87722	0.02766	25.89659
N	2	-0.28508	1.99918	5.25885	0.02705	7.28508
N	3	-0.28613	1.99918	5.26011	0.02684	7.28613
N	4	-0.28854	1.99918	5.26206	0.02729	7.28854
N	5	-0.28613	1.99918	5.26011	0.02684	7.28613
C	6	0.38644	1.99921	3.59414	0.02022	5.61356
C	7	0.37738	1.99921	3.60312	0.02029	5.62262
C	8	0.38024	1.99921	3.60037	0.02018	5.61976
C	9	0.38024	1.99921	3.60038	0.02018	5.61976
C	10	0.37738	1.99921	3.60313	0.02029	5.62262
C	11	0.38644	1.99921	3.59414	0.02022	5.61356
C	12	0.38588	1.99921	3.59462	0.02030	5.61412
C	13	0.38587	1.99921	3.59462	0.02030	5.61413
N	14	-0.39451	1.99935	5.38049	0.01467	7.39451
N	15	-0.39686	1.99935	5.38281	0.01471	7.39686
N	16	-0.39451	1.99935	5.38050	0.01467	7.39451
N	17	-0.39686	1.99935	5.38281	0.01471	7.39686
C	18	-0.08008	1.99904	4.06554	0.01550	6.08008

C	19	-0.08009	1.99904	4.06554	0.01550	6.08009
C	20	-0.08288	1.99904	4.06835	0.01548	6.08288
C	21	-0.07731	1.99904	4.06271	0.01555	6.07731
C	22	-0.08170	1.99904	4.06710	0.01556	6.08170
C	23	-0.08169	1.99904	4.06709	0.01556	6.08169
C	24	-0.07731	1.99904	4.06271	0.01555	6.07731
C	25	-0.08287	1.99904	4.06835	0.01548	6.08287
C	26	-0.17018	1.99913	4.15917	0.01187	6.17018
C	27	-0.17017	1.99913	4.15917	0.01187	6.17017
C	28	-0.16698	1.99913	4.15600	0.01185	6.16698
C	29	-0.17067	1.99913	4.15966	0.01188	6.17067
C	30	-0.16735	1.99913	4.15636	0.01186	6.16735
C	31	-0.16735	1.99913	4.15637	0.01186	6.16735
C	32	-0.17067	1.99913	4.15966	0.01188	6.17067
C	33	-0.16698	1.99913	4.15599	0.01185	6.16698
C	34	-0.20731	1.99926	4.19623	0.01182	6.20731
H	35	0.23660	0.00000	0.76154	0.00186	0.76340
C	36	-0.20731	1.99926	4.19624	0.01182	6.20731
H	37	0.23660	0.00000	0.76154	0.00186	0.76340
C	38	-0.20916	1.99926	4.19808	0.01182	6.20916
H	39	0.23701	0.00000	0.76113	0.00186	0.76299
C	40	-0.20344	1.99926	4.19241	0.01177	6.20344
H	41	0.23740	0.00000	0.76074	0.00186	0.76260
C	42	-0.20623	1.99926	4.19518	0.01179	6.20623
H	43	0.23719	0.00000	0.76095	0.00186	0.76281
C	44	-0.20623	1.99926	4.19517	0.01179	6.20623
H	45	0.23719	0.00000	0.76095	0.00186	0.76281
C	46	-0.20344	1.99926	4.19241	0.01177	6.20344
H	47	0.23740	0.00000	0.76074	0.00186	0.76260
C	48	-0.20916	1.99926	4.19808	0.01182	6.20916
H	49	0.23701	0.00000	0.76113	0.00186	0.76299
H	50	0.22359	0.00000	0.77517	0.00124	0.77641
H	51	0.22359	0.00000	0.77516	0.00124	0.77641
H	52	0.22418	0.00000	0.77458	0.00124	0.77582
H	53	0.22398	0.00000	0.77479	0.00124	0.77602
H	54	0.22420	0.00000	0.77456	0.00124	0.77580
H	55	0.22420	0.00000	0.77456	0.00124	0.77580

H	56	0.22398	0.00000	0.77478	0.00124	0.77602
H	57	0.22418	0.00000	0.77458	0.00124	0.77582
O	58	-0.09032	1.99993	6.08380	0.00658	8.09032
F	59	-0.38609	1.99998	7.38374	0.00237	9.38609
* Total *		-0.00000	101.95884	204.33746	0.70370	307.00000

NATURAL POPULATIONS: Natural atomic orbital occupancies

NAO	Atom	No	lang	Type(AO)	Occupancy
1	Fe	1	S	Cor(1S)	2.00000
2	Fe	1	S	Cor(2S)	1.99999
3	Fe	1	S	Cor(3S)	1.99646
4	Fe	1	S	Val(4S)	0.29347
5	Fe	1	S	Ryd(5S)	0.00131
6	Fe	1	S	Ryd(6S)	0.00056
7	Fe	1	px	Cor(2p)	2.00000
8	Fe	1	px	Cor(3p)	1.99854
9	Fe	1	px	Val(4p)	0.25203
10	Fe	1	px	Ryd(5p)	0.00050
11	Fe	1	py	Cor(2p)	2.00000
12	Fe	1	py	Cor(3p)	1.99858
13	Fe	1	py	Val(4p)	0.25181
14	Fe	1	py	Ryd(5p)	0.00051
15	Fe	1	pz	Cor(2p)	2.00000
16	Fe	1	pz	Cor(3p)	1.99813
17	Fe	1	pz	Val(4p)	0.23164
18	Fe	1	pz	Ryd(5p)	0.00205
19	Fe	1	dxy	Val(3d)	1.98468
20	Fe	1	dxy	Ryd(4d)	0.00330
21	Fe	1	dxy	Ryd(5d)	0.00045
22	Fe	1	dxz	Val(3d)	1.46848
23	Fe	1	dxz	Ryd(4d)	0.00682
24	Fe	1	dxz	Ryd(5d)	0.00014
25	Fe	1	dyz	Val(3d)	1.22864

26	Fe	1	dyz	Ryd(4d)	0.00286
27	Fe	1	dyz	Ryd(5d)	0.00009
28	Fe	1	dx2y2	Val(3d)	1.04734
29	Fe	1	dx2y2	Ryd(4d)	0.00312
30	Fe	1	dx2y2	Ryd(5d)	0.00003
31	Fe	1	dz2	Val(3d)	1.11913
32	Fe	1	dz2	Ryd(4d)	0.00582
33	Fe	1	dz2	Ryd(5d)	0.00009

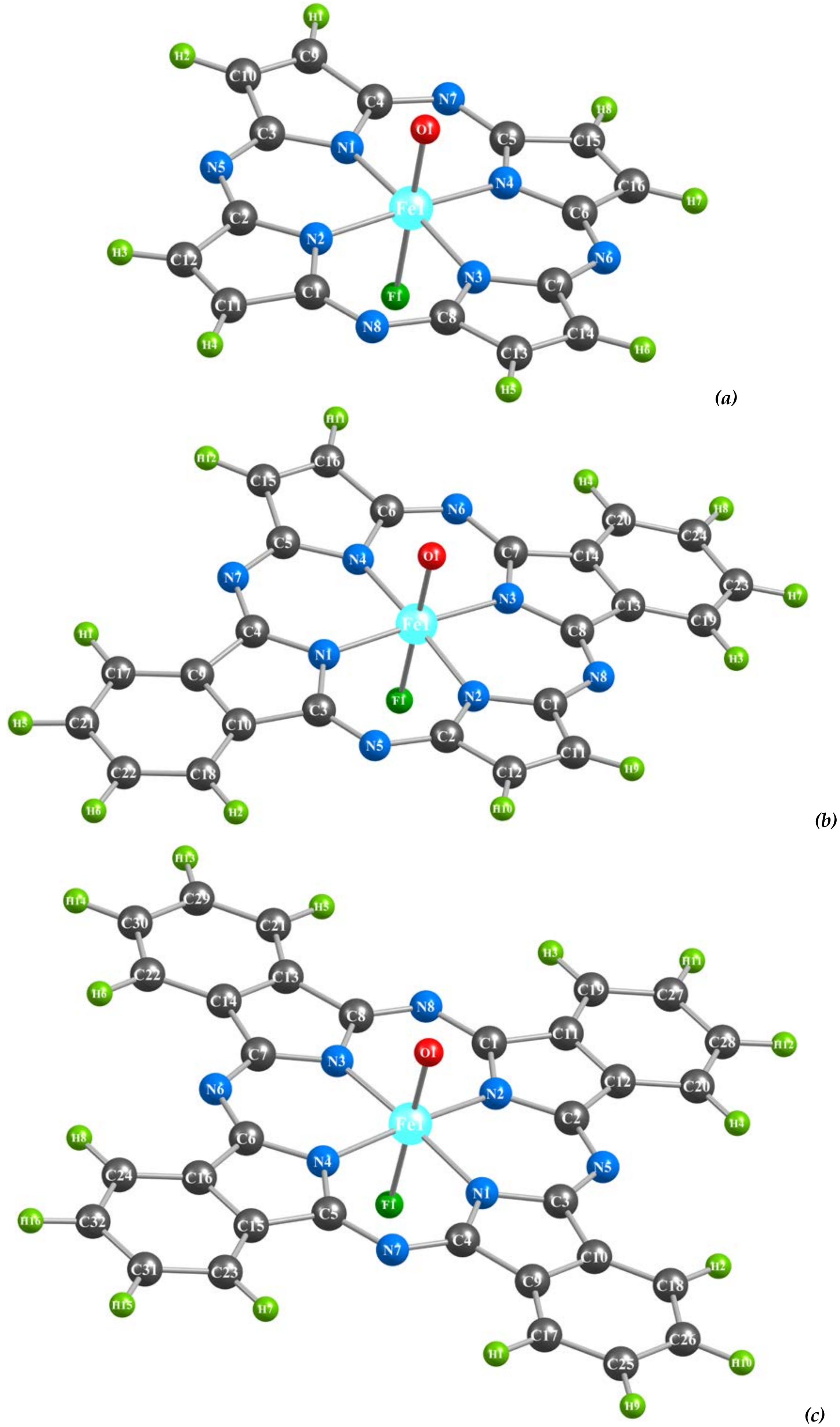

Figure *S1*. Molecular structures of the iron complexes of types **I-III** obtained by using quantum-chemical calculation by DFT OPBE/TZVP method: ***a***: [Fe**L1**(O)F], ***b***: [Fe**L2**(O)F], ***c***: [Fe**L3**(O)F].

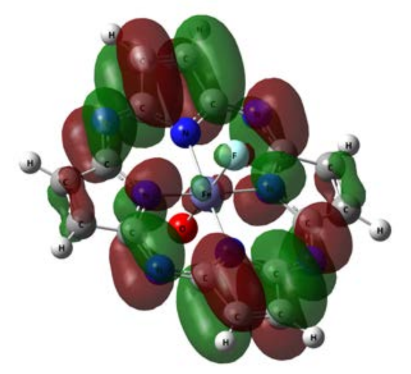	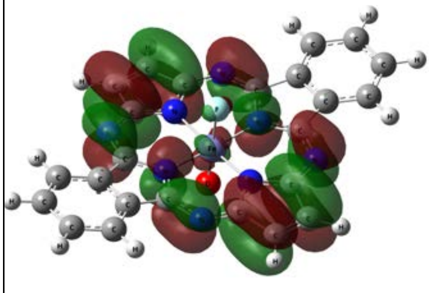	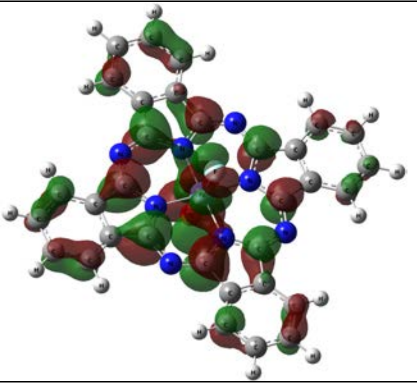
LUMO (alpha)<br>(**-4.091**)	LUMO (alpha)<br>(**-3.982**)	LUMO (beta)<br>(**-3.714**)
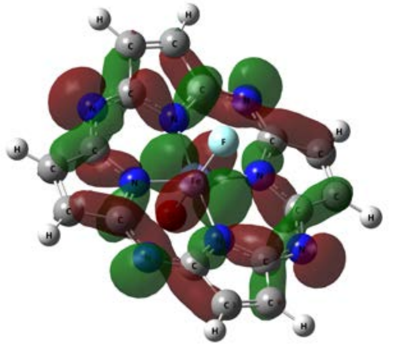	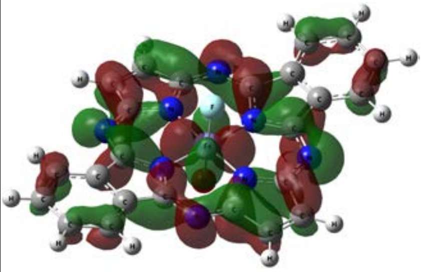	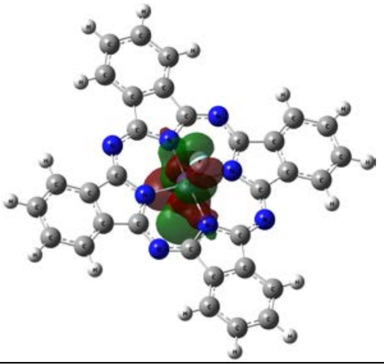
LUMO (beta)<br>(**-5.634**)	LUMO (beta)<br>(**-5.232**)	LUMO (alpha)<br>(**-4.991**)
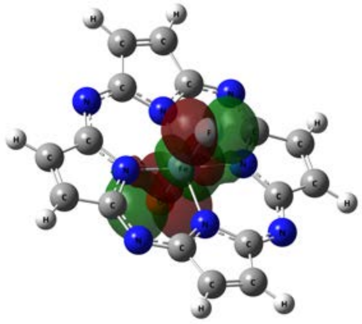	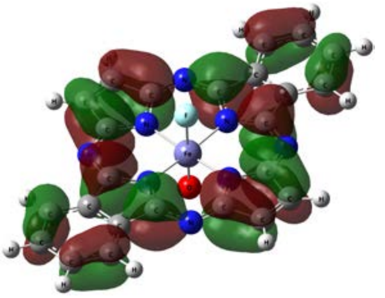	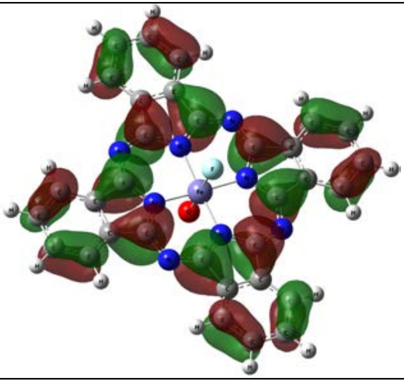
HOMO (alpha)<br>(**-5.895**)	HOMO (alpha)<br>(**-5.425**)	HOMO (alpha)<br>(**-4.993**)
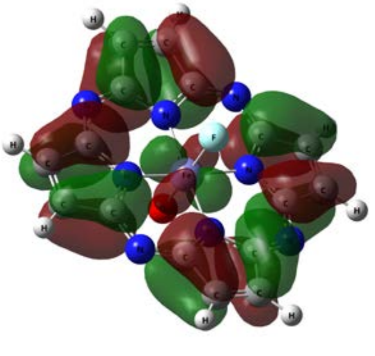	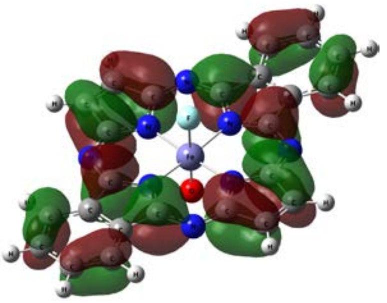	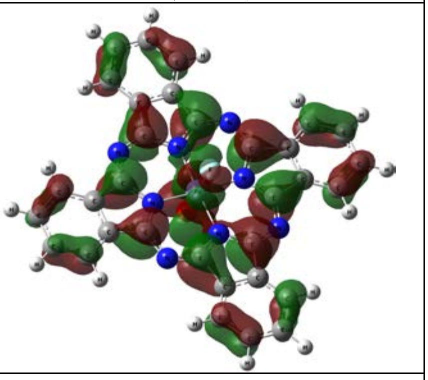
HOMO (beta)<br>(**-6.018**)	HOMO (beta)<br>(**-5.537**)	HOMO (beta)<br>(**-5.174**)
[Fe**L1**(O)F]	[Fe**L2**(O)F]	[Fe**L3**(O)F]

Figure *S2*. The pictures of HOMO and LUMO in the Fe**L1**(O)F] and [Fe**L2**(O)F] (ground state – spin quartet, M_S = 4), and [Fe**L3**(O)F] (ground state – spin doublet, M_S= 4) according to the DFT OPBE/TZVP method. The energies values of the given MOs (in brackets) are expressed in eV. The symbol “alpha” belongs to electron having spin (+1/2), symbol “beta”, to electron having spin (-l/2).
